# The Footprint of Genome Architecture in the Largest Genome Expansion in RNA Viruses

**DOI:** 10.1371/journal.ppat.1003500

**Published:** 2013-07-18

**Authors:** Chris Lauber, Jelle J. Goeman, Maria del Carmen Parquet, Phan Thi Nga, Eric J. Snijder, Kouichi Morita, Alexander E. Gorbalenya

**Affiliations:** 1 Department of Medical Microbiology, Leiden University Medical Center, Leiden, The Netherlands; 2 Department of Medical Statistics and Bioinformatics, Leiden University Medical Center, Leiden, The Netherlands; 3 Department of Virology, Institute of Tropical Medicine, Global COE Program, Nagasaki University, Nagasaki, Japan; 4 Department of Virology, National Institute of Hygiene and Epidemiology, Hanoi, Vietnam; 5 Faculty of Bioengineering and Bioinformatics, Lomonosov Moscow State University, Moscow, Russia; University of California, San Francisco, United States of America

## Abstract

The small size of RNA virus genomes (2-to-32 kb) has been attributed to high mutation rates during replication, which is thought to lack proof-reading. This paradigm is being revisited owing to the discovery of a 3′-to-5′ exoribonuclease (ExoN) in nidoviruses, a monophyletic group of positive-stranded RNA viruses with a conserved genome architecture. ExoN, a homolog of canonical DNA proof-reading enzymes, is exclusively encoded by nidoviruses with genomes larger than 20 kb. All other known non-segmented RNA viruses have smaller genomes. Here we use evolutionary analyses to show that the two- to three-fold expansion of the nidovirus genome was accompanied by a large number of replacements in conserved proteins at a scale comparable to that in the Tree of Life. To unravel common evolutionary patterns in such genetically diverse viruses, we established the relation between genomic regions in nidoviruses in a sequence alignment-free manner. We exploited the conservation of the genome architecture to partition each genome into five non-overlapping regions: 5′ untranslated region (UTR), open reading frame (ORF) 1a, ORF1b, 3′ORFs (encompassing the 3′-proximal ORFs), and 3′ UTR. Each region was analyzed for its contribution to genome size change under different models. The non-linear model statistically outperformed the linear one and captured >92% of data variation. Accordingly, nidovirus genomes were concluded to have reached different points on an expansion trajectory dominated by consecutive increases of ORF1b, ORF1a, and 3′ORFs. Our findings indicate a unidirectional hierarchical relation between these genome regions, which are distinguished by their expression mechanism. In contrast, these regions cooperate bi-directionally on a functional level in the virus life cycle, in which they predominantly control genome replication, genome expression, and virus dissemination, respectively. Collectively, our findings suggest that genome architecture and the associated region-specific division of labor leave a footprint on genome expansion and may limit RNA genome size.

## Author Summary

RNA viruses include many major pathogens. The adaptation of viruses to their hosts is facilitated by fast mutation and constrained by small genome sizes, which are both due to the extremely high error rate of viral polymerases. Using an innovative computational approach, we now provide evidence for additional forces that may control genome size and, consequently, affect virus adaptation to the host. We analyzed nidoviruses, a monophyletic group of viruses that populate the upper ∼60% of the RNA virus genome size scale. They evolved a conserved genomic architecture, and infect vertebrate and invertebrate species. Those nidoviruses that have the largest known RNA genomes uniquely encode a 3′-to-5′exoribonuclease, a homolog of canonical DNA proof-reading enzymes that improves their replication fidelity. We show that nidoviruses accumulated mutations on par with that observed in the Tree of Life for comparable protein datasets, although the time scale of nidovirus evolution remains unknown. Extant nidovirus genomes of different size reached particular points on a common trajectory of genome expansion. This trajectory may be shaped by the division of labor between open reading frames that predominantly control genome replication, genome expression, and virus dissemination, respectively. Ultimately, genomic architecture may determine the observed genome size limit in contemporary RNA viruses.

## Introduction

Genome size is the net result of evolution driven by the environment, mutation, and the genetics of a given organism [1], [2]. Particularly mutation rate is a powerful evolutionary factor [3]. The relation between mutation rate and genome size is inversely proportional for a range of life forms from viroids to viruses to bacteria, and it is positive for eukaryotes, suggestive of a causative link [4]–[6]. The genome size of RNA viruses is restricted to a range of ∼2 to 32 kb that corresponds to a very narrow band on the genome size scale (ranging from 1 kb to 10 Mb) across which genome size increase is correlated with mutation rate decrease [7]. This restricted genome size range of RNA viruses was believed to be a consequence of the universal lack of proof-reading factors, resulting in a low fidelity of RNA replication [8], [9].

In the above relation, mutation rate and proof-reading serve as a proxy for replication fidelity and genetic complexity, respectively. Replication fidelity, genome size, and genetic complexity were postulated to lock each other, through a triangular relation [10], in a low state in primitive self-replicating molecules [11]. This trapping, known as the “Eigen paradox” [12], was extended to include RNA viruses [13], providing a conceptual rationale for the small range of genome sizes in these viruses. Recent studies of the order *Nidovirales*, a large group of RNA viruses that includes those with the largest genomes known to date, provided strong support for the postulated triangular relation [10], [14]. Unexpectedly, they also revealed how nidoviruses may have solved the Eigen paradox by acquiring a proof-reading enzyme. These advancements implied that the control of genome size may be more complex than previously thought, in RNA viruses in general, and particularly in nidoviruses.

The order *Nidovirales* is comprised of viruses with enveloped virions and non-segmented single-stranded linear RNA genomes of positive polarity (ssRNA+), whose replication is mediated by a cognate RNA-dependent RNA polymerase (RdRp) [15], [16]. The order includes four families - the *Arteriviridae* and *Coronaviridae* (including vertebrate, mostly mammalian viruses), and the *Roniviridae* and *Mesoniviridae* (invertebrate viruses). The unusually broad 12.7 to 31.7 kb genome size range of this monophyletic group of viruses includes the largest known RNA genomes, which are employed by viruses from the families *Roniviridae* (∼26 kb) [17] and *Coronaviridae* (from 26.3 to 31.7 kb) [18], that have collectively been coined “large-sized nidoviruses” [19]. Viruses from the *Arteriviridae* (with 12.7 to 15.7 kb genomes) [20] and the recently established *Mesoniviridae* (20.2 kb) [21], [22] are considered small and intermediate-sized nidoviruses, respectively. Nidoviruses share a conserved polycistronic genomic architecture (known also as “organization”) in which the open reading frames (ORFs) are flanked by two untranslated regions (UTRs) [10], [23]–[26]. The two 5′-proximal ORFs 1a and 1b overlap by up to a few dozen nucleotides and are translated directly from the genomic RNA to produce polyproteins 1a (pp1a) and pp1ab, with the synthesis of the latter involving a −1 ribosomal frameshift (RFS) event [27]–[29]. The pp1a and pp1ab are autoproteolytically processed into nonstructural proteins (nsp), named nsp1 to nsp12 in arteriviruses and nsp1 to nsp16 in coronaviruses (reviewed in [30]). Most of them are components of the membrane-bound replication-transcription complex (RTC) [31]–[33] that mediates genome replication and the synthesis of subgenomic RNAs (a process known also as “transcription”) [34], [35]. ORF1a encodes proteases for the processing of pp1a and pp1ab (reviewed in [30]), trans-membrane domains/proteins (TM1, TM2, and TM3) anchoring the RTC [36]–[38] and several poorly characterized proteins. ORF1b encodes the core enzymes of the RTC (reviewed in [39], see also below). Other ORFs, whose number varies considerably among nidoviruses are located downstream of ORF1b (hereafter collectively referred to as 3′ORFs). They are expressed from 3′-coterminal subgenomic mRNAs [40], and encode virion and, optionally, so-called “accessory proteins” (reviewed in [41]–[43]). The latter, as well as several domains encoded in ORF1a and ORF1b, were implicated in the control of virus-host interactions [44]–[48].

In addition to comparable genome architectures, nidoviruses share an array (synteny) of 6 replicative protein domains. Three of these are most conserved enzymes of nidoviruses: an ORF1a-encoded protease with chymotrypsin-like fold (3C-like protease, 3CLpro) [49]–[51], an ORF1b-encoded RdRp [49], [52], [53] and a superfamily 1 helicase (HEL1) [54]–[57] (reviewed in [58]). For other proteins, relationships have been established only between some nidovirus lineages, mostly due to poor sequence similarity. Two tightly correlated properties separate large- and intermediate-sized nidoviruses from all other ssRNA+ viruses, classified in several dozens of families and hundreds of species: a genome size exceeding 20 kb and the presence of a gene encoding a RNA 3′-to-5′ exoribonuclease (ExoN), which resides in nsp14 in the case of coronaviruses [10]. The latter enzyme is distantly related to a DNA proofreading enzyme, and it is genetically segregated and expressed together with RdRp and HEL1 [14], [59]. Based on these properties ExoN was implicated in improving the fidelity of replication in large- and intermediate-sized nidoviruses. This hypothesis is strongly supported by the excessive accumulation of mutations in ExoN-defective mutants of two coronaviruses, mouse hepatitis virus [60] and severe acute respiratory syndrome coronavirus (SARS-CoV) [61], the identification of an RNA 3′-end mismatch excision activity in the SARS-CoV nsp10/nsp14 complex [62], and the high efficacy of a live coronavirus vaccine displaying impaired replication fidelity due to nsp14-knockout [63] (for review see [64], [65]). Although the molecular mechanisms underlying ExoN's function in fidelity control remain to be elucidated, its acquisition by nidoviruses likely enabled genome expansions beyond the limit observed for other non-segmented ssRNA+ viruses [10], [19]. Since ExoN-encoding nidoviruses have evolved genomes that may differ by up to ∼12 kb (from 20.2 kb of Nam Dinh virus, NDiV, to 31.7 kb of Beluga whale coronavirus SW1, BWCoV-SW1), there must be other factors in addition to the proof-reading enzyme that control genome size.

In this study we sought to characterize the dynamics of nidovirus genome expansion (NGE). The NGE is defined by the entire range of the genome sizes of extant nidoviruses, from 12.7 to 31.7 kb, and thus concerns both pre- and post-ExoN acquisition events. Our analysis revealed that ExoN acquisition was part of a larger process with non-linear dynamics, during which distinct coding regions of the nidovirus genome were expanded to accommodate both an extremely large number of mutations and virus adaptation to different host species. Our results indicate that genome architecture and the associated region-specific division of labor [1] leave a footprint on the expansion dynamics of RNA virus genomes through controlling replication fidelity and/or other mechanisms. Eventually, these constraints may determine the observed limit on RNA virus genome size.

## Results

### The scales of per-residue evolutionary change in nidoviruses and the Tree of Life are comparable

Nidoviruses have evolved genomes in a size range that accounts for the upper ∼60% of the entire RNA virus genome size scale and include the largest RNA genomes [10]. What did it take to produce this unprecedented innovation in the RNA virus world? This question could be addressed in two evolutionary dimensions: time and amount of substitutions. Due to both the lack of fossil records and high viral mutation rates, the time scale of distant relations of RNA viruses remains technically difficult to study. Hence, we sought to estimate the amount of accumulated replacements in conserved nidovirus proteins and to place it into a biological perspective by comparing it with that accumulated by proteins of cellular species in the Tree of Life (ToL).

**Figure 1:**
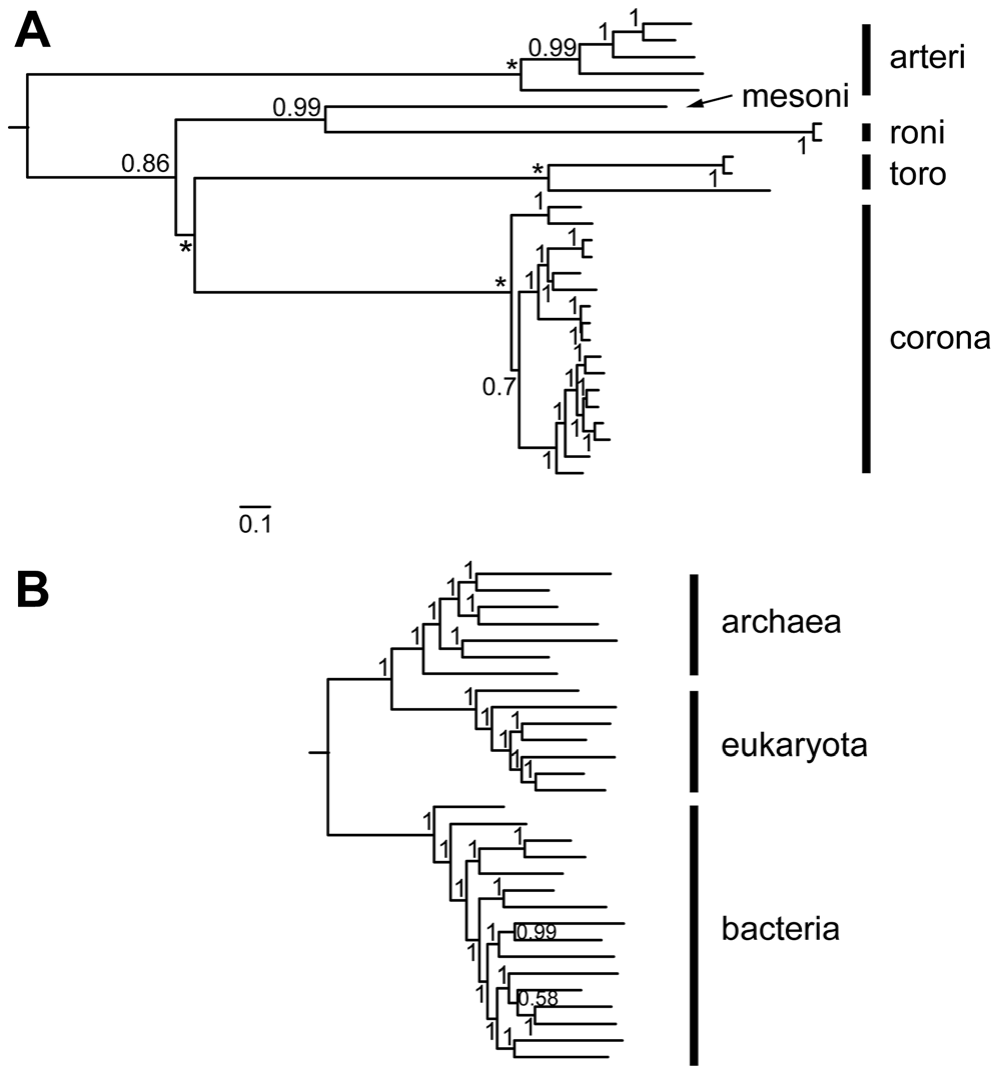
Phylogeny of nidoviruses in comparison to the Tree of life (ToL). Bayesian phylogenies of nidoviruses (A) and ToL (B) are drawn to a common scale of 0.1 amino acid substitutions per position. Major lineages are indicated by vertical bars and names; arteri: *Arteriviridae*, mesoni: *Mesoniviridae*, roni: *Roniviridae*, toro: *Torovirinae*, corona: *Coronavirinae*. Rooting was according to either (A) domain-specific outgroups [10] or (B) as described [66]. Posterior probability support values and fixed basal branch points (*) are indicated. The nidovirus and ToL alignments include, respectively, three enzymes and 56 single-gene protein families, 604 and 3336 columns, 2.95% and 2.8% gaps. For further details on the nidovirus tree see [10].

To this end, we used a rooted phylogeny for a set of 28 nidovirus representatives (Table S1), which was based on a multiple alignment of nidovirus-wide conserved protein regions in the 3CLpro, the RdRp and the HEL1, as described previously [10]. The 28 representatives covered the acknowledged species diversity of nidoviruses with completely sequenced genomes [17], [18], [20], [21] and included two additional viruses. For the arterivirus species *Porcine reproductive and respiratory syndrome virus* we selected two viruses, representing the European and North American genotypes, respectively, because we observed an unusually high divergence of these lineages; for the ronivirus species *Gill-associated virus* we selected two viruses representing the genotypes gill-associated virus and yellow head virus, respectively, because these viruses showed a genetic distance comparable to that of some coronavirus species [21] (CL & AEG, in preparation). The nidovirus-wide phylogenetic analysis consistently identified the five major lineages: subfamilies *Coronavirinae* and *Torovirinae*, and families *Arteriviridae*, *Roniviridae* and *Mesoniviridae*. The root was placed at the branch leading to arteriviruses (Fig. 1A) according to outgroup analyses [10]. Accordingly, arteriviruses with genome sizes of 12.7 to 15.7 kb are separated in the tree from other nidoviruses with larger genomes (20.2–31.7 kb).

We compared the evolutionary space explored by nidoviruses, measured in number of substitutions per site in conserved proteins, with that of a single-copy protein dataset representing the ToL [66] (Fig. 1B). Using a common normalized scale of [0,1], comparison of the viral and cellular trees and associated pairwise distance distributions revealed that the distances between cellular proteins (0.05–0.45 range) cover less than half the scale of those separating nidovirus proteins. (Fig. S1). Unlike cellular species, nidoviruses are grouped in a few compact clusters, which are very distantly related. The distances between nidovirus proteins are unevenly distributed, reflecting the current status of virus sampling: intragroup distances between nidoviruses forming major lineages are in the 0.0–0.25 range, while intergroup distances between nidoviruses that belong to different lineages are in the 0.55–1.0 range. The distances separating the intermediate-sized mesonivirus from other nidoviruses tend to be most equidistant, accounting for ∼15% of all distances in the 0.55–0.85 range. Consequently, nidovirus evolution involved the accumulation of mutations in the most conserved proteins at a scale comparable to that of the ToL. This observation is instructive in two ways. First, it can be contrasted with the conservation of nidovirus genome architecture [58], which emerges in this context as truly exceptional by conventional evolutionary considerations. Second, it makes it plausible that other, less conserved proteins might have diverged beyond the level that can be recognized by sequence alignment, thus establishing limits of the applicability of the alignment-based analysis of nidoviruses. We used both these insights to advance our study further (see below).

### The scale of nidovirus genome size change is proportional to the amount of substitutions in the most conserved proteins

**Figure 2:**
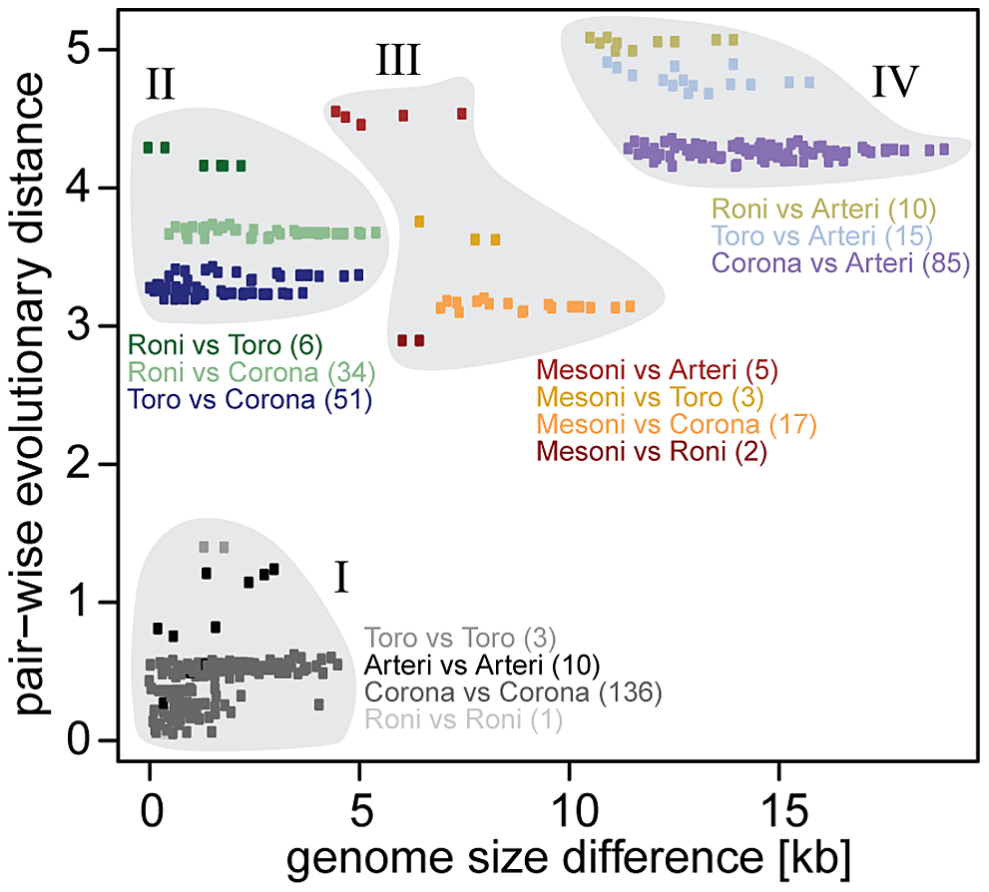
Relationship of evolutionary distance to genome size change in nidoviruses. Evolutionary distance (average number of substitutions per amino acid position in the conserved proteins) in relation to difference in genome size is shown for each pair (n = 378) of the 28 nidovirus species. Points are colored according to pairs of major clades shown in Fig. 1A. The number of comparisons for each pair of clades is indicated by numbers in brackets. Points were grouped into clusters I (intra-lineage comparisons), II (large- vs. large-sized inter-lineage comparisons), III (intermediate-sized vs. others) and IV (small- vs. large-sized).

To quantify the relation of genome size change and the accumulation of substitutions, we plotted pairwise evolutionary distances (PED) separating the most conserved replicative proteins (Y axis) versus genome size differences (X axis) for all pairs of nidoviruses in our dataset (Fig. 2). It should be noted that the observed genome size differences may serve only as a low estimate for the actual genome size change, since it does not account for (expansion or shrinkage) events that happened in parallel between two viruses since their divergence. The obtained 378 values are distributed highly unevenly, occupying the upper left triangle of the plot. Using phylogenetic considerations (Figs. 1A and S1), four clusters could be recognized in the plot. Genetic variation *within* the four major virus groups with more than one species (arteri-, corona-, roni-, and toroviruses) is confined to a compact cluster I in the left bottom corner (X range: 0.033–4.521 kb, Y range: 0.051–1.401). Values quantifying genetic divergence *between* major lineages are partitioned in three clusters taking into account genome sizes: large-sized vs. large-sized nidoviruses (cluster II, X: 0.002–5.433 kb, Y: 3.197–4.292), intermediate-sized vs. other lineages (cluster III, X: 4.475–11.494 kb, Y: 2.896–4.553), and small-sized vs. large-sized nidoviruses (cluster IV, X: 10.536–18.978 kb, Y: 4.159–5.088). Points in clusters I, III and IV are indicative of a positive proportional relation between genome size change and the accumulation of replacements. The off-diagonal location of cluster II can be reconciled with this interpretation under the (reasonable) assumption that the three lineages of large-sized nidoviruses expanded their genomes independently and considerably since diverging from their most recent common ancestor (MRCA). This positive relation is also most strongly supported by the lack of points in the bottom-right corner of the plot (large difference in genome size; small genetic divergence). Overall, this analysis indicates that a considerable change in genome size in nidoviruses could have been accomplished only when accompanied by a large number of substitutions in the most conserved proteins.

### Only a fraction of genome size changes may be attributed to known domain gain or loss

**Figure 3:**
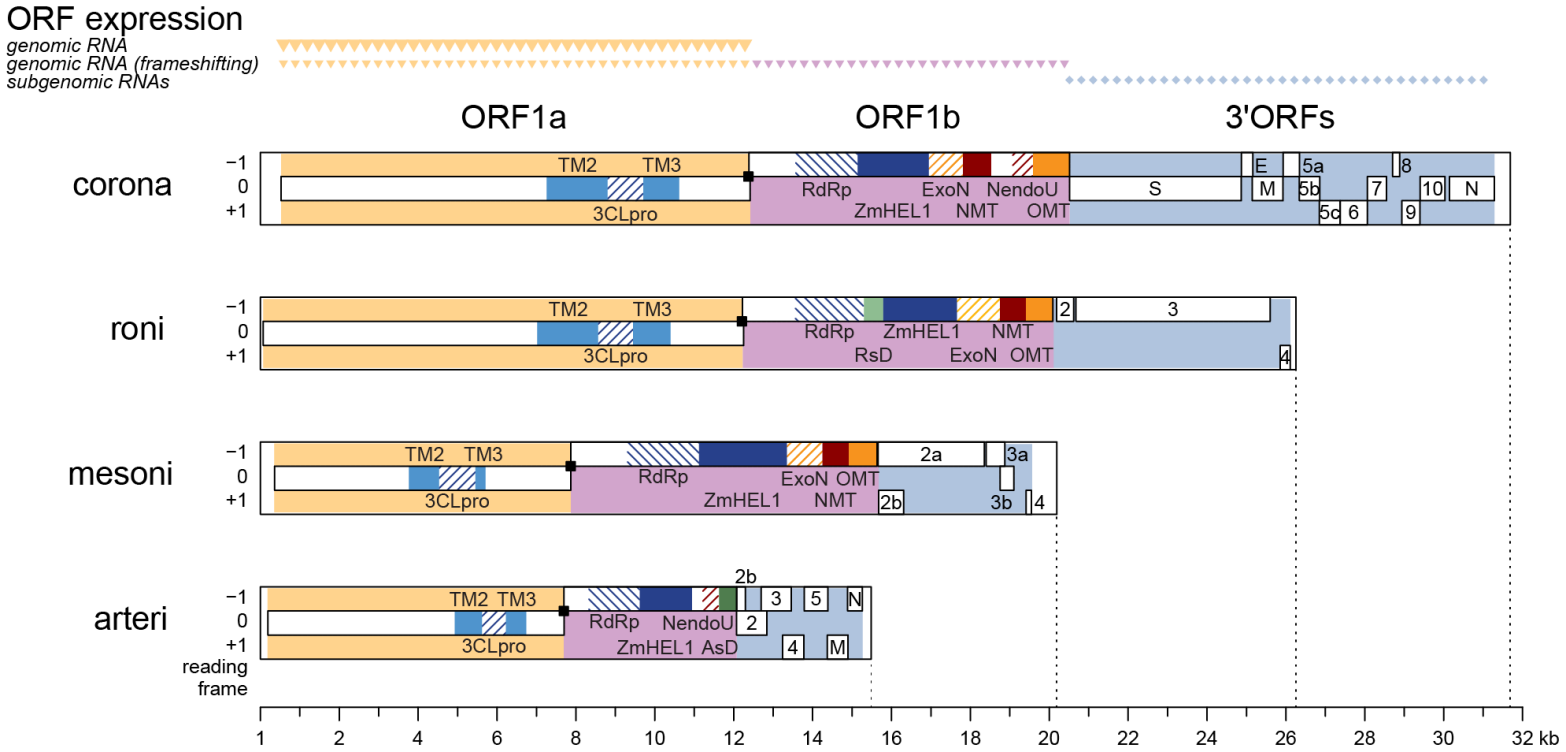
Genomic organization and expression, and key domains of four nidoviruses. The coding regions are partitioned into ORF1a (yellow), ORF1b (violet) and the 3′ORFs (blue), which also differ in expression mechanism as indicated on top. Black squares, ribosomal frameshifting sites. Within ORFs (white rectangles), colored patterns highlight domains identified in: all nidoviruses [TM2, TM3, 3CLpro, RdRp, and Zn-cluster binding domain fused with HEL1 (ZmHEL1) [114] - light and dark blue], large nidoviruses (ExoN, OMT - orange), certain clades (NMT, NendoU - red; ronivirus-specific domain (RsD) - light green; arterivirus-specific domain (AsD) - dark green). Genomic organizations are shown for Beluga whale coronavirus SW1 (corona), gill-associated virus (roni), Nam Dinh virus (mesoni), and porcine respiratory and reproductive syndrome virus North American type (arteri).

Next, we asked whether genome size change could be linked to domain gain and loss. We analyzed the phylogenetic distribution of protein domains that were found to be conserved in one or more of the five major nidovirus lineages [10]. Ancestral state parsimonious reconstruction was performed for the following proteins: ORF1b-encoded ExoN, N7-methyltransferase (NMT) [67], nidovirus-specific endoribonuclease (NendoU) [68], [69], 2′-O-methyltransferase (OMT) [70], [71], ronivirus-specific domain (RsD) (this study; see legend to Fig. S2), and ORF1a-encoded ADP-ribose-1″-phosphatase (ADRP) [72]–[74]. This analysis revealed that domain gain and loss have accompanied NGE (Fig. S2 and Table S2). Particularly, the genetically segregated ExoN, OMT and NMT domains (Fig. 3) were acquired in a yet-to-be determined order during the critical transition from small-sized to intermediate-sized nidovirus genomes. However, the combined size of these domains [10] accounts for only a fraction (49.7%) of the size difference (4,475 nt) between the genomes of NDiV (20,192 nt) and Simian hemorrhagic fever virus (SHFV), which has the largest known arterivirus genome (15,717 nt). The fraction that could be attributed to these and the three other protein domains is even smaller in other pairs of viruses representing different major nidovirus lineages (CL & AEG). This analysis is also complicated by the uncertainty about the genome sizes of nidovirus ancestors that acquired or lost domains.

### The nidovirus genome can be partitioned according to functional conservations in genome architecture

In order to gain further insight in NGE dynamics, we analyzed large genome areas in which homology signals were not recoverable in the currently available dataset because of both the extreme divergence of distant nidoviruses and the relatively poor virus sampling (Fig. 1). To address this challenge, we developed an approach that establishes and exploits relationships between nidovirus genomes in an alignment-free manner on grounds other than sequence homology. To this end, we partitioned the nidovirus genome according to functional conservations in the genome architecture, using results for few characterized nidoviruses and bioinformatics-based analysis for most other viruses (reviewed in [19]). With this approach, the genomes of all nidoviruses can be consistently partitioned into five regions in the 5′ to 3′ order: 5′-UTR, ORF1a, ORF1b, 3′ORFs, and 3′-UTR (Fig. 3, Table S3). The 5′-UTR and 3′-UTR flank the coding regions and account for <5% of the nidovirus genome size. The borders of the three ORF regions that overlap by few nucleotides in some or all nidoviruses were defined as follows: ORF1a: from the ORF1a initiation codon to the RFS shifty codons; ORF1b: from the RFS signal to the ORF1b termination codon; and 3′ORFs: from the ORF1b termination codon to the termination codon of the ORF immediately upstream of the 3′UTR. As we detail in the Supplementary text (Text S1), the three ORF regions are of similar size but differ in expression mechanism (Fig. 3 top) and principal function. Thus, ORF1a dominates the expression regulation of the entire genome, and ORF1b encodes the principal enzymes for RNA synthesis, while the 3′ORFs control genome dissemination. This region-specific association may be described as a division of labor [1].

### The nidovirus genome expanded unevenly across the three major coding regions

**Figure 4:**
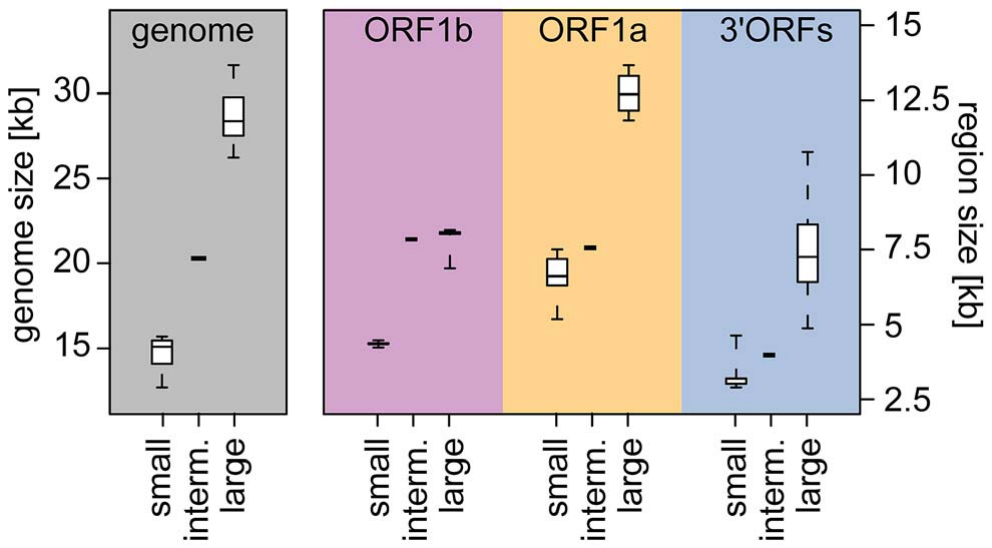
Nidovirus genome and region size differences. Shown are size distributions of genomes (left part) and the three genome coding parts ORF1b, ORF1a and 3′ORFs (right part) for five small-sized arterivirus species (small), 22 large-sized nidovirus species (large) and one intermediate-sized mesonivirus species (interm.). The distributions are represented by box-and-whisker graphs, where the box spans from the first to the third quartile and includes the median (bold line). The whiskers extend (dashed lines) to the extreme values.

**Figure 5:**
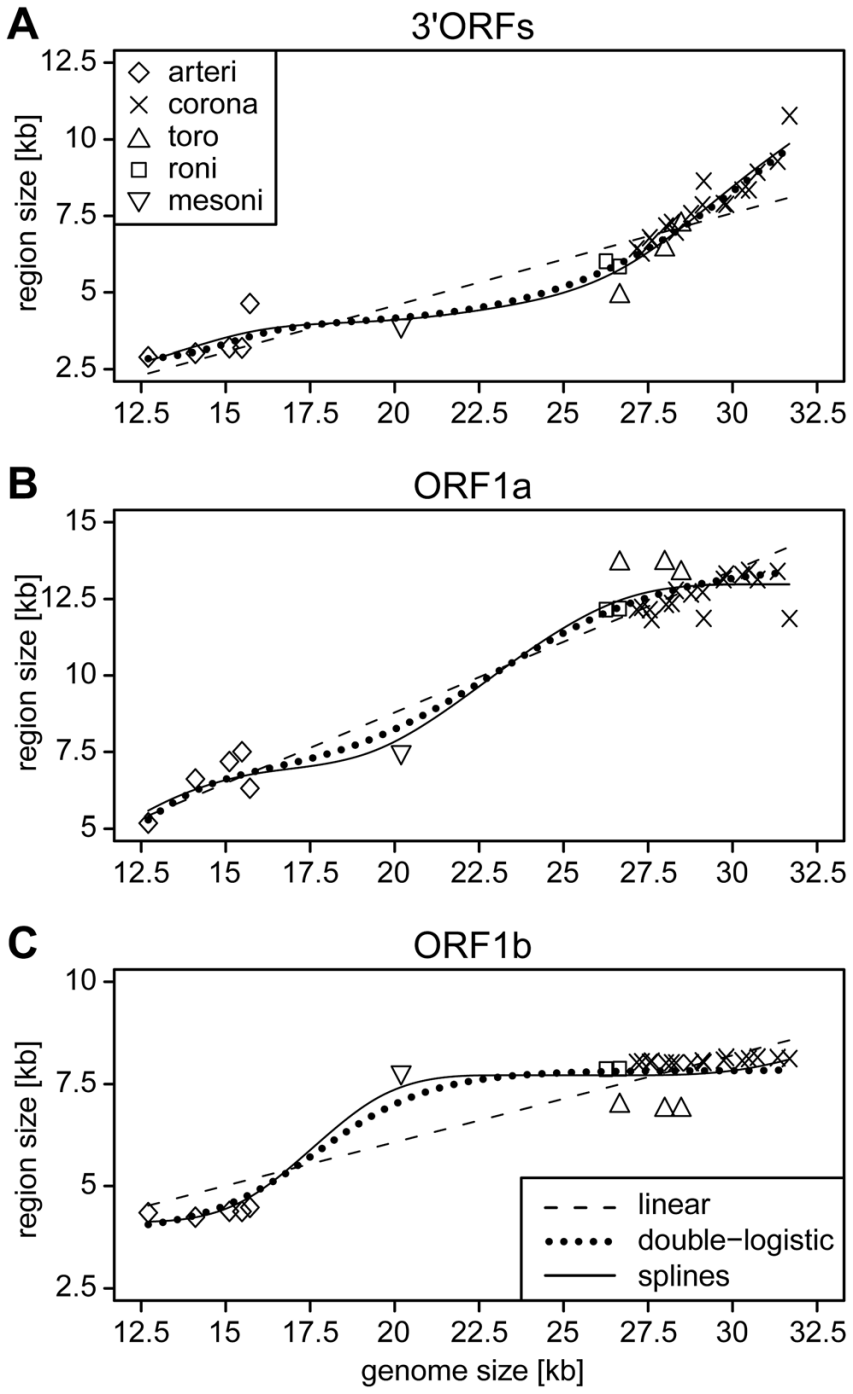
Relationship of sizes of three major coding regions and genome size in the nidovirus evolution. For 28 nidoviruses representing species diversity, absolute sizes of 3′ORFs (A), ORF1a (B), and ORF1b (C) are plotted against the size of the genome. Different symbols were used to group the viruses into five major phylogenetic lineages (see inlet in A). Results of weighted linear, double-logistic and 3rd order monotone splines [111] regression analyses are depicted. The three regression models (see inlet in C) fit the data with weighted r^2^ values of 0.908 (linear), 0.949 (double-logistic) and 0.960 (splines) for ORF1a, 0.758, 0.898 and 0.929 for ORF1b, and 0.835, 0.950 and 0.954 for 3′ORFs. For fit comparison of regression models see Table 1.

We then asked how the different regions contributed to the genome expansion. We initially noted that the intermediate position of the mesonivirus between the two other nidovirus groups is observed only in genome-wide but not in region-specific size comparisons (Fig. 4). In the latter, the mesonivirus clusters with either small-sized (ORF1a and 3′ORFs) or large-sized (ORF1b) nidoviruses. This non-uniform position of the mesonivirus relative to other nidoviruses is indicative of a non-linear relationship between the size change of the complete genome and its individual regions during NGE. Accordingly, when fitting weighted linear regressions for the three regions separately to the six datasets formed by nidoviruses with small and large genomes, support for a linear relationship was found only for the 3′ORF dataset of large nidoviruses; for all other regions a linear relationship was not statistically significant (Fig. S3). These results prompted us to evaluate linear as well as non-linear regression models applied to a dataset including all known nidovirus species (n = 28) (Fig. 5). Two non-linear models were employed: third order monotone splines and a double-logistic regression. In the monotone splines, two parameters – the number and position of knots – determine the regression fit. We identified values for both parameters that result in the best fit (Fig. S4).

Using weighted r^2^ values, we observed that the splines model captures 92.9–96.1% of the data variation for the three ORF regions. This was a 5–22% gain in the fit compared to the linear model (75.9–90.8%) (Fig. 5). This gain was considered statistically significant (α = 0.05) in two F-tests, a specially designed one and a standard one, as well as in the LV-test for every ORF region (p = 0.019 or better) and, particularly, their combination (p = 9.1e-6 or better) (Table 1). The splines model also significantly outperforms the double-logistic model (p = 0.0014) (Table 1). These results established that the nidovirus genome expanded in a non-linear and region-specific fashion.

**Table 1:** Comparison of regression models.

comparison^a^		test^b^	regression statistics^c^			
*model A*	*model B*		*ORF1a*	*ORF1b*	*3′ORFs*	*total*
linear	splines	F	0.0190*	0.0009*	0.0005*	1.8e-8*
linear	splines	F_perm_	0.0009*	0.0036*	<1.0e-6*^d^	1.0e-6*
linear	splines	LV	0.0032*	0.0065*	0.0049*	9.1e-6*
linear	dlog	LV	0.0011*	0.0100*	0.0035*	8.5e-6*
dlog	splines	LV	0.0300*	0.0019*	0.2196	1.4e-3*

a linear regression model (linear); double-logistic regression model (dlog); 3^rd^ order monotone splines regression model (splines).

b standard weighted F test (F); permutation F test (F_perm_); a weighted version of a test to compare non-nested regression models (LV) as described in [112].

c shown is the probability that model A (null hypothesis) fits the data better than model B (alternative hypothesis); asterisks (*) highlight significant values to reject the null in favor of the alternative hypothesis using a confidence level of 0.05; probabilities are calculated separately for ORF1a, ORF1b, 3′ORFs as well as the complete model combining the three coding plus the two UTR regions (total).

d none of the 1 million permutations resulted in an F larger than that of the non-permuted dataset.

### The three major coding regions expanded consecutively in a lineage-dependent manner

**Figure 6:**
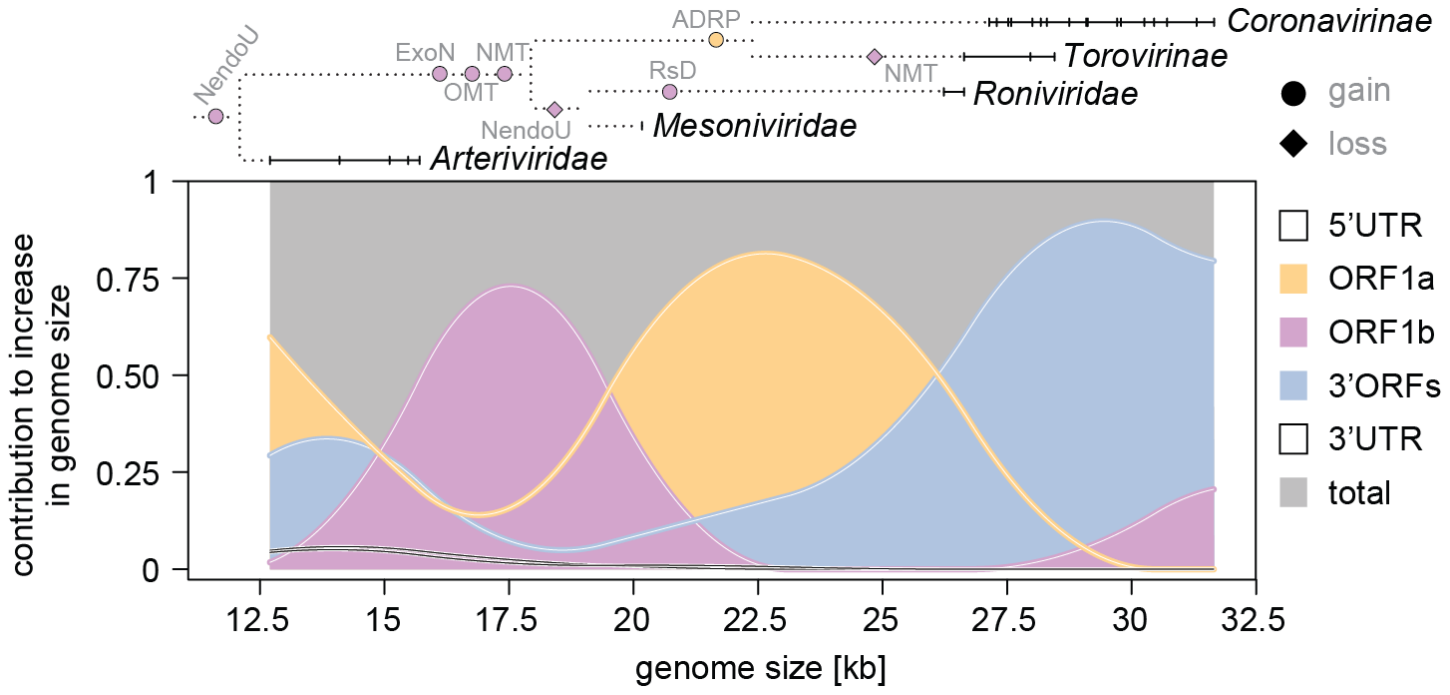
Region-specific, wavelike dynamics of the nidovirus genome expansions. Relative contributions of the genome regions ORF1a, ORF1b, 3′ORFs, 5′UTR and 3′UTR to the increase in genome size are calculated according to the splines regression and plotted on top of each other and against their sum = 1. Solid horizontal lines and vertical bars on top: genome size ranges and samplings for nidovirus lineages indicated by names. Dotted lines: topology of major nidovirus branches. Selected domains gained (ExoN, OMT, NMT, RsD and ADRP, circles) and lost (NendoU and NMT, diamonds) are colored according to ORF in which they are encoded. See also Fig. 3, Fig. S2 and the main text.

Like each region, also the entire genome must have expanded non-linearly during NGE. Revealing its dynamic was our next goal. To this end, we analyzed the contribution of each of the five genomic regions to the overall genome size increase under the three models (Fig. 6 and Fig. S5). The top-ranking splines model (Table 1) predicts a cyclic pattern of overlapping wavelike size increases for the three coding regions (the 5′ and 3′UTR account only for a negligibly minor increase that is limited to small nidoviruses). Each of the three coding regions was found to have increased at different stages during NGE (Fig. 6). A cycle involves expanding predominantly and consecutively the ORF1b, ORF1a, and 3′ORFs region. One complete cycle flanked by two partial cycles are predicted to have occurred during the NGE from small-sized to large-sized nidoviruses. The complete cycle encompasses almost the entire genome size range of nidoviruses, starting from 12.7 kb and ending at 31.7 kb. The dominance of an ORF region in the increase of genome size was characterized by two parameters: a genome size range (X axis in Fig. 6) in which the contribution of a region accounts for a >50% share of the total increase, and by the maximal share it attains in the NGE (Y axis in Fig. 6). For three major regions these numbers are: ORF1b, dominance in the 15.8–19.3 kb range with 72.9% maximal contribution at genome size 17.5 kb; ORF1a, 19.7–26.1 kb and 81.3% at 22.7 kb; 3′ORFs, 26.1–31.7 kb and 89.6% at 29.5 kb (Fig. 6).

Furthermore, the shapes of the three waves differ. The first one (ORF1b) is most symmetrical and it starts and ends at almost zero contribution to the genome size change. This indicates that the ORF1b expansion is exceptionally constrained, which is in line with the extremely narrow size ranges of ORF1b in arteri- and coronaviruses (with mean±s.d. of 4362±86 and 8073±50 nt, respectively; Fig. 4 and Fig. 6). The second wave (ORF1a) is tailed at the upper end and is connected to the ORF1a wave from the prior cycle. This ORF seems to have a relatively high baseline contribution (∼20%) to the genome size change up to the range of coronaviruses. The third wave (3′ORFs) is most asymmetrical (incomplete), as it only slightly decreases from its peak toward the largest nidovirus genome size to which this region remains the dominant contributor (∼77%).

One partial cycle, preceding the complete one, is observed inside the genome size range of arteriviruses and involves the consecutive expansions of ORF1a and 3′ORFs, respectively. Also the main, but still very limited contributions of 5′- and 3′-UTRs (<6%) are observed here. The start of another incomplete cycle, involving the expansion of ORF1b and overlapping with the complete cycle, is observed within the upper end of coronavirus genome sizes.

It must be stressed that nidoviruses occupy different positions on the trajectory that depicts the entire NGE dynamics. For the viruses with large genomes those with smaller genomes represent stages that they have passed during NGE; in this respect the latter may resemble ancestral viruses which have gone extinct. For the smaller genomes those with the larger ones represent stages that they have not reached during NGE. Mesonivirus and roniviruses seem to have been “frozen” after the first (ORF1b) and second (ORF1a) wave, respectively. The third wave (3′ORFs) was due to the genome expansion of coronaviruses and, to a lesser extent, toroviruses (compare the genome sizes of these viruses and the third wave position in Fig. 6). These observations reveal that the constraints on genome size due to genome architecture may be modulated in a lineage-dependent manner.

## Discussion

In this study we provide, for the first time, a quantitative insight into the large-scale evolutionary dynamics of genome expansion in RNA viruses that concerns the upper ∼60% of the RNA virus genome size scale exclusively populated by nidoviruses. In view of the extremely large amount of substitutions that accumulated in the nidovirus genome during evolution, we exploited the functional conservation in the nidovirus genome architecture to partition genomes of nidoviruses into five non-overlapping regions. Using a complex statistical framework, we discovered that consecutive, region-specific size increases must have occurred during NGE. We conclude that the genome size dynamics in nidoviruses may be shaped by the division of labor between ORFs that predominantly control genome replication, genome expression, and virus dissemination, respectively.

Genome size evolution in RNA viruses, unlike that of DNA-based life forms, has received relatively little attention from the research community. The small range of RNA genome sizes might have been perceived as evidence for the lack of meaningful genome size dynamics in RNA viruses. Even if there was any dynamics, its reconstruction could be considered a challenging if not impossible task, since evolutionary signals of distant relationships would not be recoverable, possibly due to the saturation of the genome with substitutions [9], [75]. To our knowledge, genome size increase in RNA viruses has thus far been associated with only a few trends: a concomitant increase of the average size of replicative proteins [76], a reduction of genome compression as measured by gene overlaps [77], and a strong correlation between the presence of helicase [19], [78] and ExoN [10], [14] domains and the genome size in ssRNA+ viruses.

Now, by analyzing NGE, we show that even in the most conserved proteins genome expansion was accompanied by a considerable accumulation of replacements, which may approach saturation (Fig. 2). In other, less conserved proteins this effect is expected to be (much) larger. That relation is in line with the observation that nucleotide substitutions are on average four times more common than insertions/deletions in RNA viruses [7]. Practically, this result indicates that even for the study of a large monophyletic group like the nidoviruses, the power of substitution-based (phylogenetic) analysis is limited. We have overcome this limitation by employing an innovative approach that exploits functional conservation in genome architecture rather than sequence homology. The inferred non-linear dynamics of NGE is supported strongly by different statistical tests. However, in view of the highly uneven distribution of genomes sizes in our dataset, which may be considered a problem, we will provide additional supporting arguments below.

First of all, we note that a virus (called Cavally virus) that is closely related to the unique intermediate-sized NDiV was independently identified in a parallel study [26]. Both viruses share all properties that are critical for this study, including the size of genome and ORFs as well as the assignment of protein domains [21]. These results show that the NDiV characteristics used in our study are reliable. Second, these two mesoniviruses and the very distant roniviruses, which have large genomes, form a monophyletic group (Fig. 1). This clustering correlates with common (molecular) properties, including the infection of invertebrate hosts and the lack of the NendoU domain, which distinguish mesoni- and roniviruses from other vertebrate nidoviruses (Fig. S2) and may apply to other yet-to-be identified members of this group as well. Third, even if we restrict our analysis to small- and large-sized nidoviruses, differences between the size ranges of genomes versus the three ORF regions are already apparent (Fig. 4). Particularly striking are the extremely constrained size of ORF1b in both arteriviruses and coronaviruses as well as the exceptionally large size range of 3′ORFs in large-sized nidoviruses. These constraints contribute prominently to the first and third wave, respectively, of the major NGE cycle (Fig. 6). Thus, the described dynamics of the region-specific genome size increase reflects properties of both mesoniviruses and other nidoviruses, and is expected to be sustained while virus sampling continues.

Poor virus sampling limits the resolution of our reconstruction of domain gain/loss during NGE. For instance, the critically important acquisition of ExoN seems to be tightly correlated with those of two replicative methyltransferases, NMT and OMT (Fig. S2). The fact that NMT and ExoN are adjacent domains of a single protein in coronaviruses (nsp14) whereas OMT resides nearby (nsp16) in pp1ab suggests a link between these domains and indicates that NMT and ExoN may have been acquired in a single event. Furthermore, NMT and OMT were shown to be essential for cap formation at the 5′-end of coronavirus mRNAs [67], [70], [71], with the OMT-mediated modification proposed to be important for the evasion of innate immunity [47]. These enzymes are yet to be characterized in other large-sized nidoviruses.

The ExoN acquisition is a hallmark of the first NGE wave because it is expected to have improved the replication fidelity and, thus, made further genome expansion feasible. In contrast, no domain acquisition with a comparably strong biological rationale could be identified for the second wave. Two aspects, both contrasting the first and second wave, are noteworthy. Firstly, while the first wave seems to reflect genome expansion in a *single* ancestral lineage that might have given rise to all intermediate- and large-sized nidoviruses (founding event), the second wave encompasses expansions in *several* lineages that happened in *parallel* (Fig. S2B). Secondly, evolutionary relations of ORF1a-encoded proteins (underlying the second wave) are not as extensively documented as those for ORF1b (underlying the first wave), since ORF1a proteins in nidoviruses have diverged to a far greater extent. Hence, the domain gain/loss description for the second wave is even less complete than that for the first wave. Most notable is the acquisition of ADRP (formerly termed “X domain” [79]), whose physiological function remains elusive (see Supplementary text S2) and which seems to be part of the second wave in large-sized vertebrate nidoviruses (Fig. 6). Unlike the first and second wave, the third one encompasses changes that predominantly happened during the radiation of a subfamily (*Coronavirinae*) rather than several families (Fig. 6); they are being analyzed in a separate study (CL & AEG, in preparation). Improved future virus sampling, especially in the genome size range around 20 kb, could be critical for the description of domain gain/loss in ORF1a and its refinement in ORF1b (Fig. S2).

**Figure 7:**
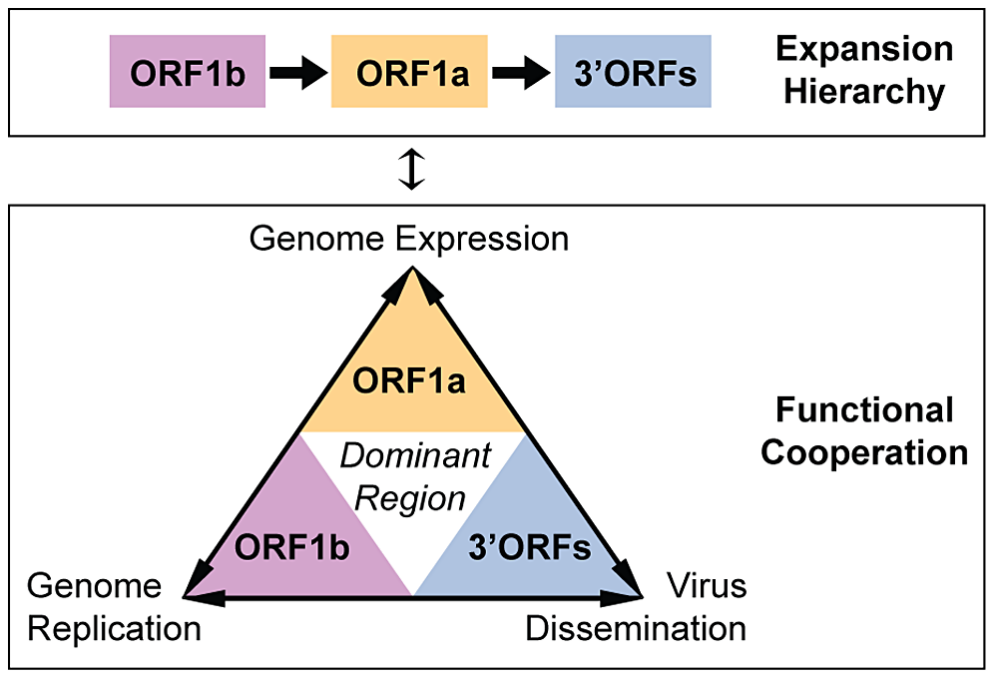
Hierarchy and cooperation in the nidovirus genome expansions. Functional and evolutionary relations between the three major coding regions of the nidovirus genome are depicted. For a brief description on the relationship between these three coding regions and the processes they dominate in the nidovirus life cycle, see text.

Products of ORF1b, ORF1a, and 3′-ORFs, whose expansion dynamics are reported here, cooperate bidirectionally in the nidovirus life cycle [15], since their functioning depends on each other (Fig. 7, bottom). In contrast, the order in which these regions expanded is unidirectional (Fig. 7 top). It implies a causative chain of events during NGE and suggests, for the first time and unexpectedly, a hierarchy of the three underlying biological processes. To our knowledge, no theory or results published provided a basis for the model describing how genome expansion must have proceeded. Now that the dynamics of NGE have been established, it could be further rationalized using experimental data on the functions of the proteins involved. Importantly and regardless of how plausible these functional considerations might sound, they do not substitute for the evidence of the inferred dynamics presented elsewhere in the paper.

The association of the first wave of domain acquisitions with ORF1b may attest to the universal critical role of replicative enzymes in NGE beyond the 20-kb threshold observed for other ssRNA+ viruses (for discussion see [10]). Regardless in which order the *OMT*, *NMT* and *ExoN* loci were acquired, their products must have been adapted to the core of the RTC that is formed by the ORF1b-encoded RdRp and HEL1-containing proteins [53], [58], [80]. Other, less conserved RTC components are encoded in ORF1a [34], [37], [70], [81]–[83]. It is known that proteins encoded in ORF1a and ORF1b interact in coronaviruses [34], [84], [85] and likely arteriviruses [86], [87]. Some of these interactions, e.g. between nsp10 and nsp14 or nsp16, were shown to be essential for the function of the ORF1b-encoded enzymes [62], [88], [89]. Accordingly, the RTC, already enlarged with the newly acquired ORF1b-encoded subunits, could have triggered and/or become accommodative of the expansion of ORF1a. Additionally, the ORF1a expansion may have been prompted by the need to adapt the expression mechanisms it controls to the changes of the ORF1b-encoded part that had already increased in size and complexity. The final wave of expansion involving the 3′ORFs may have been triggered by the need for virus particle adaptation to accommodate the expanded genome. This plausible link was extensively explored in the literature that implicated packaging head space in the control of genome size in other viruses [90]–[94]). The sizes of genomes and virus particles may also be correlated in nidoviruses, although the evolution of virion size in nidovirus lineages has not been studied to our knowledge. During NGE, a part of the newly acquired genetic material may have been adapted to facilitate both virus-host interactions [46], [48], [95], [96] and coordination between the three ORF regions for the benefit of the processes they control and the life cycle [97]. For instance, in arteriviruses the ORF1a-encoded nsp1 is essential for subgenomic mRNA synthesis and virion biogenesis [86], [87], [98] and a role in transcription was proposed for an ORF1a-encoded domain of nsp3 in coronaviruses [99]. Thus, factors encoded by ORF1a and ORF1b might constrain NGE by controlling the expression of the 3′ORFs region and/or the functioning of its products. This would explain why the 3′ORFs expansion could not have been possible *before* the expansion of ORF1a and ORF1b. Based on a similar line of reasoning, an extremely tight control of the ORF1b size (Fig. 4) may set the ultimate NGE size limit.

The order in which the three coding regions expanded matches their ranking in terms of sequence conservation, which is evident from the distribution of nidovirus conserved domains across these regions (Figs. 2 and 3). This conservation is inversely proportional to the amount of accumulated substitutions, although a quantitative characterization of the latter aspect is yet to be systematically documented. Genome changes due to region-specific expansion and residue substitution may affect each other, and both may contribute to virus adaptation to the host. In this respect we noticed that viruses with larger genomes, compared to their small-sized cousins, may employ a larger repertoire of proteins for interacting with the host. It is also apparent that large-sized nidoviruses may afford both the acquisition and loss of an ORF as a matter of genome variation in a species (see e.g. [100]–[102]; for review see [103]). Thus, large genomes could provide nidoviruses with an expanded toolkit to adapt upon crossing species barriers and to explore new niches in established hosts.

### Concluding remarks and implications

It is broadly acknowledged that high mutation rates and large population sizes allow RNA viruses to explore an enormous evolutionary space and to adapt to their host [76], [104]. Yet the low fidelity of replication also confines their evolution within a narrow genome size range that must affect their adaptation potential. Above, we present evidence for a new constraint on genome size in RNA viruses. In our analysis of nidoviruses, the conserved genome architecture and associated division of labor emerged as potentially powerful forces that are involved in selecting both new genes and positions of gene insertion during genome expansion. In this respect, the established wavelike dynamics of regional size increase could be seen as the footprint of genome architecture on genome size evolution. Ultimately, these constraints may determine the upper limit of the RNA virus genome size. The reported data point to an important evolutionary asymmetry during genome expansion, which concerns the relation between proteins controlling genome replication, expression, and dissemination, and may certainly be relevant beyond the viruses analyzed here.

Importantly, the major diversification of nidoviruses by genome expansion must have started at some early point after the acquisition of ExoN [10]. From that point on, nidoviruses expanded their genomes in parallel in an increasing number of lineages, each of which may have acquired different domains in the same region. Extant representatives of the major lineages have very different genome sizes and essentially offer snapshots of different NGE stages. It seems that the host range may affect the outcome of this process, since the two families that infect invertebrates are on the lower end of the genome size range in the ExoN-encoding nidoviruses. For yet-to-be described nidoviruses, the genome expansion model can predict the sizes of the three coding regions by knowing the genome size only. The mechanistic basis of this fundamental relation can be probed by comparative structure-function analyses, which may also advance the development of nidovirus-based vectors and rational measures for virus control. Thus, the wavelike dynamics model links virus discovery to basic research and its various applications.

## Methods

### Datasets

A dataset of nidoviruses representing species diversity from the three established and a newly proposed virus family was used (Table S1). A multiple alignment of nidovirus-wide conserved protein domains (28 species, 3 protein families, 604 aa alignment positions, 2.95% gap content) as described previously [10] formed the basis of all phylogenetic analyses. To put the scale of the nidovirus evolution into an independent perspective, we compared it with a cellular dataset previously used to reconstruct the ToL, for which a concatenated alignment of single-copy proteins was used (30 species, 56 protein families, 3336 aa alignment positions, 2.8% gap content) [66]. The proteins used in the nidoviral and cellular datasets are the most conserved in their group and, as such, could be considered roughly equivalent and suitable for the purpose of this comparative analysis.

### Phylogenetic analyses

Rooted phylogenetic reconstructions by Bayesian posterior probability trees utilizing BEAST [105] under the WAG amino acid substitution matrix [106] and relaxed molecular clock (lognormal distribution) [107] were performed as described previously [10]. Evolutionary pairwise distances were calculated from the tree branches. A maximum parsimony reconstruction of the ancestral nidovirus protein domain states at internal nodes of the nidovirus tree was conducted using PAML4 [108].The quality of ancestral reconstructions was assessed by accuracy values provided by PAML4. The nidovirus genomic sequences are non-independent due to their phylogenetic relatedness [109]. When calculating the contribution of individual sequences to the total observed genetic diversity the uneven sampling of different phyletic lineages must be accounted for. To correct for the uneven sampling we assigned relative weights to the 28 nidovirus species by using position-based sequence weights [110] that were calculated on the alignment submitted for phylogeny reconstruction. The weights were normalized to sum up to one and were used in regression analyses (see below). The sequence weights varied ∼7 fold from 0.017 to 0.116. NDiV, which represents mesoniviruses, showed the largest weight of 0.116 that was distantly followed by those of the bafinivirus White bream virus (WBV; 0.075) and roniviruses (0.06 each); coronaviruses, making up the best-sampled clade, were assigned the lowest weights (0.017 to 0.028 each).

### Statistical analysis of genome size change in nidoviruses

The genome of each nidovirus was consistently partitioned into five genomic regions according to external knowledge (see Results). To model the contribution of each genomic region to the total genome size change, we conducted weighted regression analyses (size of a genomic region on size of the genome) using three models – a linear and two non-linear ones. Position-based sequence weights were used and a confidence level of α = 0.05 was applied in all analyses. The regressions of the different genomic regions were not fitted separately but were joined to produce a genome-wide analysis. The combined contribution of all genomic regions to the genome size change must obviously sum up to 100%. To satisfy this common constraint, in each analysis, regression functions were fitted simultaneously to sizes of the genomic regions by minimizing the residual sum of squares, thereby constraining the sum of all slopes to be not larger than one. The linear model assumes a constant contribution of each genomic region during evolution which was modeled via linear regions.

In the first non-linear model we applied third order monotone splines with equidistant knots [111]. We chose splines because of their flexibility and generality (we do not rely on a specific regression function). The monotonicity constraint was enforced to avoid overfitting which was observed otherwise, and third order functions were chosen to obtain smooth, second-order derivatives. We explored the dependence of the performance of the splines model on variations in two critical parameters, the number of knots and the start position of the first knot. These two parameters define a knot configuration and determine a partitioning of the data into bins. In the first test we evaluated five different configurations generating from three to seven knots. Configurations using eight or more knots resulted in some bins being empty and were therefore not considered. For each number of knots the position of the first knot and the knot distance were determined as resulting in that configuration for which the data points are distributed most uniformly among the resulting bins. The exception was the 3-knot configuration, in which the position of the second knot was selected as the intermediate position in the observed genome size range (22.2 kb). Only configurations with equidistant knots were considered. All probed splines models were evaluated by goodness-of-fit values (weighted version of the coefficient of determination r^2^). In the second test we evaluated the model dependence on the position of the first knot by considering all positions that do not result in empty bins for the optimal number of knots determined using the approach described above.

As another non-linear model we used a 7-parameter double-logistic regression function that mimics the splines model and more readily allows for biological interpretations. Logistic functions discriminate between two principal states – stationary and growth phases; a double-logistic curve comprises not more than three steady and two growth phases. The “length” of the different phases (in the dimension of the independent variable; e.g. genome size), the steady state values (in the dimension of the dependent variable, e.g. ORF size), and the “strength” of the growth (e.g. the maximum slope of the curve between two steady states) are controlled by the parameters of the regression function. Once estimated, the parameter values can be used to infer genome size intervals for which a particular ORF region is in a steady state as well as critical genome and ORF sizes at the transition between two steady states. Since double-logistic regressions did not converge for the 5′- and 3′-UTRs, linear functions were used for these two genome regions instead.

Linear (null hypothesis) and splines (alternative hypothesis) regression models were compared using standard weighted F-statistics and a specially designed permutation test (see below). To exclude overfitting as the cause of support of the more complex models, we utilized a more sophisticated framework (LV-Test) for the comparison of non-nested regression models (linear vs. double-logistic and splines vs. double-logistic) as detailed in [112]. The test was further modified to include weighted residuals according to virus sequence weights that account for sequence dependence.

Since our null hypothesis (linear model) is at the boundaries of the parameter space, we developed a permutation test to further compare the linear and splines models. To this end, genome region sizes were transformed to proportions (region size divided by genome size), randomly permuted relative to genome sizes, and transformed back to absolute values. These transformations are compatible with the constraints of the null hypothesis and the requirement that region sizes have to sum to genome sizes. Weights were not permuted. The linear and splines models were fit to the permuted datasets and F-statistics were calculated as for the original dataset. The p-value of the test is the fraction of F-statistics of permuted datasets that are larger than the F of the original dataset. It was calculated using 1,000,000 permutations that were randomly sampled out of ∼10^29^ possible permutations.

Finally, we analyzed the contribution of each genome region to the total change in genome size under the three regression models. The contribution of each region according to a model was calculated as the ratio of change in region size to change in genome size (first derivative of the regression function) along the nidovirus genome size scale. These region-specific contributions were combined in a single plot for visualization purposes.

To conduct all statistical analyses and to visualize the results we used the R package [113].

### Accession numbers

Accession numbers of virus genomes utilized in the study are shown in Table S1.

## Supporting Information

Figure S1 **Comparison of genetic distances of nidoviruses and cellular organisms.** Shown are the distributions of pair-wise distances for nidovirus and cellular single-copy conserved proteins according to the phylogenies in Fig. 1. The combined set of distances was normalized relative to the largest distance that was set to one.
(TIF)

Figure S2 **Gain and loss of selected ORF1a/ORF1b domains found in subsets of nidoviruses.** (A) Distribution of six selected domains identified in ORF1a (one) and ORF1b (five) conserved in subsets of 28 nidovirus species (right part). One of the ORF1b-encoded domains (RsD) was identified in this study by inspection of the pp1b alignment as a ronivirus-specific insertion (163 aa) that is located between the conserved RdRp and ZmHEL1 domains (see Fig. 3). Colors indicate a domain's ORF location (purple for ORF1b, yellow for ORF1a). The left part shows predicted gain (circles colored according to its ORF location) and loss (colored diamonds) events at internal branches of the nidovirus phylogeny [10]. Nidovirus ancestral domain compositions were reconstructed utilizing a maximum parsimony analysis implemented in PAML4. Support values are shown in Table S2. (B) The nidovirus phylogeny was mapped on the genome size scale (dotted lines). Individual genome sizes of 28 nidovirus species are shown by vertical dashes and the size range within major lineages by horizontal solid lines. Internal nodes in the tree were arbitrarily placed at half the distance of adjacent branching events connecting two lineages while observing the original topology of the phylogeny. Predicted domain gain/loss events are highlighted as in (A).
(TIF)

Figure S3 **Clade-specific relationship of sizes of three major coding regions and genome size in the nidovirus evolution.** For 28 nidoviruses representing species diversity, absolute sizes of 3′ORFs (A), ORF1a (B), and ORF1b (C) are plotted against the size of the genome. Different symbols were used to group the viruses into five major phylogenetic lineages (see inlet in A). Results of weighted linear regression analyses for small-sized (arteri) and large-sized nidoviruses (corona, toro/bafini, roni) are depicted. Regressions with a slope significantly different from zero are shown in black, non-significant ones in grey. The linear regressions fit the data with p = 0.11, r2 = 0.63 (arteri) and p = 0.51, r2 = 0.03 (corona, toro/bafini, roni) for ORF1a, p = 0.34, r2 = 0.30 and p = 0.08, r2 = 0.23 for ORF1b, and p = 0.22, r2 = 0.44 and p = 1e-10, r2 = 0.89 for 3′ORFs. The only significant correlation was observed for 3′ORFs of nidoviruses with large genomes (A) where the regression line showed a slope of 0.84 (±0.07 s.e.).
(TIF)

Figure S4 **Sensitivity of the splines regression model to the number of knots and the position of the first knot.** Shown are goodness-of-fit in form of weighted r2 values (A–C, G–I) and sensitivity on the resulting regression curve (D–F, J–L) for different number of knots in the range of 3 to 7 (A–F) and different positions of the first knot (G–L) for the 3′ORFs, ORF1a and ORF1b genome regions. The best fit was obtained for the 7-knot configuration for all three regions (A–C). Hence, the 7-knot configuration was selected as the optimal one. We have also calculated a difference between other splines models compared to the optimal knot number by calculating the absolute difference of the regression curves of two configurations normalized to the size range of observed values (e.g. size ranges of ORF1a, ORF1b or 3′ORFs). This difference was in the range of 1–7% and increased with decreasing knot number in all three regions (D–F); it could be viewed as the loss of fit relative to the 7-knot configuration. Also, we calculated the model dependence on the position of the first knot by evaluating all positions that do not result in empty bins for the 7-knot configuration, which was found to be in the range from 11.4 to 12.0 kb (G–I). There was virtually no dependence of the position of the first knot and the goodness-of-fit (G–L); we selected the position that is closest to the minimal genome size. The knot number (k = 7) and position of the first knot (at 12 kb resulting in a knot distance of 3.7 kb) used in the main calculation are indicated by green vertical lines.
(TIF)

Figure S5 **Modeling contribution of ORF1a, ORF1b, 3′ORFs, 5′UTR and 3′UTR to the nidovirus genome expansion.** Relative contributions of ORF1a (yellow), ORF1b (purple), 3′ORFs (blue), and 5′ and 3′UTR (black) to the increase in genome size are plotted on top of each other and against their sum = 1 (grey) for the linear (A), the splines (B) and the doublelogistc (C) regression model. Relative size contributions were calculated based on the regression curves fitted to the five genome parts for a dataset of 28 nidoviruses representing species diversity. Solid horizontal lines and vertical bars on top: genome size ranges and virus samplings for arteri-, corona-, toro-/bafini-, roni- and mesoniviruses. Under the linear model (which was statistically rejected in favor of the non-linear models), the contribution of each region to the genome size change is constant by definition. The ORF1a region accounts for most change (46.3%), followed by 3′ORFs (30.2%), ORF1b (21.3%), 5′UTR (1.3%) and 3′UTR (0.9%). In contrast, the splines and double-logistic models predict a cyclic pattern of overlapping wavelike increases of sizes for the three ORFs regions, with maximal contributions of 72.9%, 81.3% and 89.6% for ORF1b, ORF1a and 3′ORFs, respectively (see also main text). Highly similar cyclic and wave-like patterns of region expansions are predicted by the double-logistic model that mostly differs in the amplitude and range of waves compared to those of the splines model. These similarities suggest that the double-logistic model might be an approximation of the monotone splines model facilitating biologically meaningful interpretations.
(TIF)

Table S1 **Nidovirus representatives.**
(DOC)

Table S2 **Nidovirus ancestral protein domain reconstruction.**
(DOC)

Table S3 **Dataset of region and genome sizes used in this study.**
(RTF)

Text S1 **Division of labor in the nidovirus genome.**
(DOCX)

Text S2 **ADRP acquisition and second wave of genome expansion in coronaviruses.**
(DOCX)

## References

[1] Szathmary E, Smith JM (1995) The Major Evolutionary Transitions. Nature 374: 227–232. 7885442 10.1038/374227a0

[2] Lynch M, Conery JS (2003) The origins of genome complexity. Science 302: 1401–1404. 14631042 10.1126/science.1089370

[3] Drake JW, Charlesworth B, Charlesworth D, Crow JF (1998) Rates of spontaneous mutation. Genetics 148: 1667–1686. 9560386 10.1093/genetics/148.4.1667 PMC1460098

[4] Sniegowski PD, Gerrish PJ, Johnson T, Shaver A (2000) The evolution of mutation rates: separating causes from consequences. Bioessays 22: 1057–1066. 11084621 10.1002/1521-1878(200012)22:12<1057::AID-BIES3>3.0.CO;2-W

[5] Gago S, Elena SF, Flores R, Sanjuan R (2009) Extremely High Mutation Rate of a Hammerhead Viroid. Science 323: 1308. 19265013 10.1126/science.1169202

[6] Lynch M (2010) Evolution of the mutation rate. Trends Genet 26: 345–352. 20594608 10.1016/j.tig.2010.05.003 PMC2910838

[7] Sanjuan R, Nebot MR, Chirico N, Mansky LM, Belshaw R (2010) Viral Mutation Rates. J Virol 84: 9733–9748. 20660197 10.1128/JVI.00694-10 PMC2937809

[8] Steinhauer DA, Domingo E, Holland JJ (1992) Lack of Evidence for Proofreading Mechanisms Associated with An Rna Virus Polymerase. Gene 122: 281–288. 1336756 10.1016/0378-1119(92)90216-c

[9] Holmes EC (2011) What Does Virus Evolution Tell Us about Virus Origins? J Virol 85: 5247–5251. 21450811 10.1128/JVI.02203-10 PMC3094976

[10] Nga PT, Parquet MD, Lauber C, Parida M, Nabeshima T, et al (2011) Discovery of the First Insect Nidovirus, a Missing Evolutionary Link in the Emergence of the Largest RNA Virus Genomes. PLoS Pathog 7: e1002215. 21931546 10.1371/journal.ppat.1002215 PMC3169540

[11] Eigen M (1971) Selforganization of Matter and Evolution of Biological Macromolecules. Naturwissenschaften 58: 465–523. 4942363 10.1007/BF00623322

[12] Kun A, Santos M, Szathmary E (2005) Real ribozymes suggest a relaxed error threshold. Nat Genet 37: 1008–1011. 16127452 10.1038/ng1621

[13] Holmes, E C. (2009) The Evolution and Emergence of RNA Viruses. New York: Oxford University Press. 254 p.

[14] Snijder EJ, Bredenbeek PJ, Dobbe JC, Thiel V, Ziebuhr J, et al (2003) Unique and conserved features of genome and proteome of SARS-coronavirus, an early split-off from the coronavirus group 2 lineage. J Mol Biol 331: 991–1004. 12927536 10.1016/S0022-2836(03)00865-9 PMC7159028

[15] Perlman S., Gallagher T., and Snijder, E J., Eds. (2008) Nidoviruses. Washington, DC: ASM Press.

[16] de Groot RJ, Cowley JA, Enjuanes L, Faaberg KS, Perlman S, et al. (2012) Order *Nidovirales*. In: King AMQ, Adams MJ, Carstens EB, Lefkowitz EJ, editors. Virus Taxonomy, Ninth Report of the International Committee on Taxonomy of Viruses. Amsterdam: Elsevier Academic Press. pp. 785–795.

[17] Cowley JA, Walker PJ, Flegel TW, Lightner DV, Bonami JR, et al. (2012) Family *Roniviridae*. In: King AMQ, Adams MJ, Carstens EB, Lefkowitz EJ, editors. Virus Taxonomy, Ninth Report of the International Committee on Taxonomy of Viruses. Amsterdam: Elsevier Academic Press. pp. 829–834.

[18] de Groot RJ, Baker SC, Baric R, Enjuanes L, Gorbalenya AE, et al. (2012) Family *Coronaviridae*. In: King AMQ, Adams MJ, Carstens EB, Lefkowitz EJ, editors. Virus Taxonomy, Ninth Report of the International Committee on Taxonomy of Viruses. Amsterdam: Elsevier Academic Press. pp. 806–828.

[19] Gorbalenya AE, Enjuanes L, Ziebuhr J, Snijder EJ (2006) Nidovirales: Evolving the largest RNA virus genome. Virus Res 117: 17–37. 16503362 10.1016/j.virusres.2006.01.017 PMC7114179

[20] Faaberg KS, Balasuriya UB, Brinton MA, Gorbalenya AE, Leung FC-C, et al. (2012) Family *Arteriviridae*. In: King AMQ, Adams MJ, Carstens EB, Lefkowitz EJ, editors. Virus Taxonomy, Ninth Report of the International Committee on Taxonomy of Viruses. Amsterdam: Elsevier Academic Press. pp. 796–805.

[21] Lauber C, Ziebuhr J, Junglen S, Drosten C, Zirkel F, et al (2012) Mesoniviridae: a new family in the order *Nidovirales* formed by a single species of mosquito-borne viruses. Arch Virol 157: 1623–1628. 22527862 10.1007/s00705-012-1295-x PMC3407358

[22] Adams MJ, King AMQ, Carstens EB (2013) Ratification vote on taxonomic proposals to the International Committee on Taxonomy of Viruses (2013). Arch Virol 158: 1181–8 doi:10.1007/s00705-013-1688-5 23580178 10.1007/s00705-013-1688-5

[23] Boursnell MEG, Brown TDK, Foulds IJ, Green PF, Tomley FM, et al (1987) Completion of the sequence of the genome of the coronavirus avian infectious bronchitis virus. J Gen Virol 68: 57–77. 3027249 10.1099/0022-1317-68-1-57

[24] den Boon JA, Snijder EJ, Chirnside ED, de Vries AA, Horzinek MC, et al (1991) Equine arteritis virus is not a togavirus but belongs to the coronaviruslike superfamily. J Virol 65: 2910–2920. 1851863 10.1128/jvi.65.6.2910-2920.1991 PMC240924

[25] Cowley JA, Walker PJ (2002) The complete genome sequence of gill-associated virus of Penaeus monodon prawns indicates a gene organization unique among nidoviruses. Arch Virol 147: 1977–1987. 12376758 10.1007/s00705-002-0847-x PMC7086798

[26] Zirkel F, Kurth A, Quan PL, Briese T, Ellerbrok H, et al (2011) An Insect Nidovirus Emerging from a Primary Tropical Rainforest. mBio 2: e00077–11. 21673192 10.1128/mBio.00077-11 PMC3111606

[27] Brierley I, Boursnell ME, Binns MM, Bilimoria B, Blok VC, et al (1987) An efficient ribosomal frame-shifting signal in the polymerase-encoding region of the coronavirus IBV. EMBO J 6: 3779–3785. 3428275 10.1002/j.1460-2075.1987.tb02713.x PMC553849

[28] Plant EP, Perez-Alvarado GC, Jacobs JL, Mukhopadhyay B, Hennig M, et al (2005) A three-stemmed mRNA pseudoknot in the SARS coronavirus frameshift signal. PLoS Biol 3: 1012–1023. 10.1371/journal.pbio.0030172 PMC1110908 15884978

[29] Firth AE, Brierley I (2012) Non-canonical translation in RNA viruses. J Gen Virol 93: 1385–1409. 22535777 10.1099/vir.0.042499-0 PMC3542737

[30] Ziebuhr J, Snijder EJ, Gorbalenya AE (2000) Virus-encoded proteinases and proteolytic processing in the Nidovirales. J Gen Virol 81: 853–879. 10725411 10.1099/0022-1317-81-4-853

[31] Denison MR, Spaan WJ, van der Meer Y, Gibson CA, Sims AC, et al (1999) The putative helicase of the coronavirus mouse hepatitis virus is processed from the replicase gene polyprotein and localizes in complexes that are active in viral RNA synthesis. J Virol 73: 6862–6871. 10400784 10.1128/jvi.73.8.6862-6871.1999 PMC112771

[32] Shi ST, Schiller JJ, Kanjanahaluethai A, Baker SC, Oh JW, et al (1999) Colocalization and membrane association of murine hepatitis virus gene 1 products and De novo-synthesized viral RNA in infected cells. J Virol 73: 5957–5969. 10364348 10.1128/jvi.73.7.5957-5969.1999 PMC112657

[33] van Hemert MJ, van den Worm SHE, Knoops K, Mommaas AM, Gorbalenya AE, et al (2008) SARS-coronavirus replication/transcription complexes are membrane-protected and need a host factor for activity in vitro. PLoS Pathog 4: e1000054. 18451981 10.1371/journal.ppat.1000054 PMC2322833

[34] Sawicki SG, Sawicki DL, Younker D, Meyer Y, Thiel V, et al (2005) Functional and genetic analysis of coronavirus replicase-transcriptase proteins. PLoS Pathog 1: 310–322. 10.1371/journal.ppat.0010039 PMC1298938 16341254

[35] Thiel V, Herold J, Schelle B, Siddell SG (2001) Viral replicase gene products suffice for coronavirus discontinuous transcription. J Virol 75: 6676–6681. 11413334 10.1128/JVI.75.14.6676-6681.2001 PMC114390

[36] van der Meer Y, van Tol H, Locker JK, Snijder EJ (1998) ORF1a-encoded replicase subunits are involved in the membrane association of the arterivirus replication complex. J Virol 72: 6689–98. 9658116 10.1128/jvi.72.8.6689-6698.1998 PMC109868

[37] Harcourt BH, Jukneliene D, Kanjanahaluethai A, Bechill J, Severson KM, et al (2004) Identification of Severe Acute Respiratory Syndrome Coronavirus Replicase Products and Characterization of Papain-Like Protease Activity. J Virol 78: 13600–13612. 15564471 10.1128/JVI.78.24.13600-13612.2004 PMC533933

[38] Baliji S, Cammer SA, Sobral B, Baker SC (2009) Detection of Nonstructural Protein 6 in Murine Coronavirus-Infected Cells and Analysis of the Transmembrane Topology by Using Bioinformatics and Molecular Approaches. J Virol 83: 6957–6962. 19386712 10.1128/JVI.00254-09 PMC2698535

[39] Ziebuhr J (2005) The coronavirus replicase. Curr Top Microbiol Immunol 287: 57–94. 15609509 10.1007/3-540-26765-4_3 PMC7121973

[40] Sawicki SG, Sawicki DL, Siddell SG (2007) A contemporary view of coronavirus transcription. J Virol 81: 20–29. 16928755 10.1128/JVI.01358-06 PMC1797243

[41] Brian DA, Baric RS (2005) Coronavirus genome structure and replication. Curr Top Microbiol Immunol 287: 1–30. 15609507 10.1007/3-540-26765-4_1 PMC7120446

[42] Enjuanes L, Almazan F, Sola I, Zuniga S (2006) Biochemical aspects of coronavirus replication and virus-host interaction. Ann Rev Microbiol 60: 211–230. 16712436 10.1146/annurev.micro.60.080805.142157

[43] Masters PS (2006) The molecular biology of coronaviruses. Adv Virus Res 66: 193–292. 16877062 10.1016/S0065-3527(06)66005-3 PMC7112330

[44] de Groot RJ (2006) Structure, function and evolution of the hemagglutinin-esterase proteins of corona- and toroviruses. Glycoconjugate Journal 23: 59–72. 16575523 10.1007/s10719-006-5438-8 PMC7088178

[45] Frieman M, Baric R (2008) Mechanisms of Severe Acute Respiratory Syndrome Pathogenesis and Innate Immunomodulation. Microbiology and Molecular Biology Reviews 72: 672–685. 19052324 10.1128/MMBR.00015-08 PMC2593566

[46] Graham RL, Sparks JS, Eckerle LD, Sims AC, Denison MR (2008) SARS coronavirus replicase proteins in pathogenesis. Virus Res 133: 88–100. 17397959 10.1016/j.virusres.2007.02.017 PMC2637536

[47] Zust R, Cervantes-Barragan L, Habjan M, Maier R, Neuman BW, et al (2011) Ribose 2′-O-methylation provides a molecular signature for the distinction of self and non-self mRNA dependent on the RNA sensor Mda5. Nat Immunol 12: 137–143. 21217758 10.1038/ni.1979 PMC3182538

[48] Zhao L, Jha BK, Wu A, Elliott R, Ziebuhr J, et al (2012) Antagonism of the Interferon-Induced OAS-RNase L Pathway by Murine Coronavirus ns2 Protein Is Required for Virus Replication and Liver Pathology. Cell Host & Microbe 11: 607–616. 22704621 10.1016/j.chom.2012.04.011 PMC3377938

[49] Gorbalenya AE, Koonin EV, Donchenko AP, Blinov VM (1989) Coronavirus genome: prediction of putative functional domains in the non-structural polyprotein by comparative amino acid sequence analysis. Nucl Acids Res 17: 4847–4861. 2526320 10.1093/nar/17.12.4847 PMC318036

[50] Anand K, Palm GJ, Mesters JR, Siddell SG, Ziebuhr J, et al (2002) Structure of coronavirus main proteinase reveals combination of a chymotrypsin fold with an extra alpha-helical domain. EMBO J 21: 3213–3224. 12093723 10.1093/emboj/cdf327 PMC126080

[51] Barrette-Ng IH, Ng KKS, Mark BL, van Aken D, Cherney MM, et al (2002) Structure of arterivirus nsp4 - The smallest chymotrypsin-like proteinase with an alpha/beta C-terminal extension and alternate conformations of the oxyanion hole. J Biol Chem 277: 39960–39966. 12163505 10.1074/jbc.M206978200

[52] Cheng A, Zhang W, Xie Y, Jiang W, Arnold E, et al (2005) Expression, purification, and characterization of SARS coronavirus RNA polymerase. Virology 335: 165–176. 15840516 10.1016/j.virol.2005.02.017 PMC7111802

[53] te Velthuis AJW, Arnold JJ, Cameron CE, van den Worm SHE, Snijder EJ (2010) The RNA polymerase activity of SARS-coronavirus nsp12 is primer dependent. Nucl Acids Res 38: 203–214. 19875418 10.1093/nar/gkp904 PMC2800238

[54] Hodgman TC (1988) A new superfamily of replicative proteins. Nature 333: 22–23. 10.1038/333022b0 3362205

[55] Gorbalenya AE, Koonin EV, Donchenko AP, Blinov VM (1988) A novel superfamily of nucleoside triphosphate-binding motif containing proteins which are probably involved in duplex unwinding in DNA and RNA replication and recombination. FEBS Lett 235: 16–24. 2841153 10.1016/0014-5793(88)81226-2 PMC7130140

[56] Seybert A, Hegyi A, Siddell SG, Ziebuhr J (2000) The human coronavirus 229E superfamily 1 helicase has RNA and DNA duplex-unwinding activities with 5′-to-3′ polarity. RNA 6: 1056–1068. 10917600 10.1017/s1355838200000728 PMC1369980

[57] Seybert A, van Dinten LC, Snijder EJ, Ziebuhr J (2000) Biochemical characterization of the equine arteritis virus helicase suggests a close functional relationship between arterivirus and coronavirus helicases. J Virol 74: 9586–9593. 11000230 10.1128/jvi.74.20.9586-9593.2000 PMC112390

[58] Gorbalenya AE (2001) Big nidovirus genome - When count and order of domains matter. Adv Exp Med Biol 494: 1–17. 11774451

[59] Minskaia E, Hertzig T, Gorbalenya AE, Campanacci V, Cambillau C, et al (2006) Discovery of an RNA virus 3′→5′ exoribonuclease that is critically involved in coronavirus RNA synthesis. Proc Natl Acad Sci U S A 103: 5108–5113. 16549795 10.1073/pnas.0508200103 PMC1458802

[60] Eckerle LD, Lu X, Sperry SM, Choi L, Denison MR (2007) High fidelity of murine hepatitis virus replication is decreased in nsp14 exoribonuclease mutants. J Virol 81: 12135–12144. 17804504 10.1128/JVI.01296-07 PMC2169014

[61] Eckerle LD, Becker MM, Halpin RA, Li K, Venter E, et al (2010) Infidelity of SARS-CoV Nsp14-Exonuclease Mutant Virus Replication Is Revealed by Complete Genome Sequencing. PLoS Pathog 6: e1000896. 20463816 10.1371/journal.ppat.1000896 PMC2865531

[62] Bouvet M, Imbert I, Subissi L, Gluasis L, Canard B, et al (2012) RNA 3′-end mismatch excision by the severe acute respiratory syndrome coronavirus nonstructural protein nsp10/nsp14 exoribonuclease complex. Proc Natl Acad Sci U S A 109: 9372–9377. 22635272 10.1073/pnas.1201130109 PMC3386072

[63] Graham RL, Becker MM, Eckerle LD, Bolles M, Denison MR, et al (2012) A live, impaired-fidelity coronavirus vaccine protects in an aged, immunocompromised mouse model of lethal disease. Nat Med 18: 1820–1826. 23142821 10.1038/nm.2972 PMC3518599

[64] Denison MR, Graham RL, Donaldson EF, Eckerle LD, Baric RS (2011) Coronaviruses: An RNA proofreading machine regulates replication fidelity and diversity. RNA Biol 8: 270–279. 21593585 10.4161/rna.8.2.15013 PMC3127101

[65] Smith EC, Denison MR (2012) Implications of altered replication fidelity on the evolution and pathogenesis of coronaviruses. Curr Opin Virol 2: 519–524. 22857992 10.1016/j.coviro.2012.07.005 PMC7102773

[66] Boussau B, Blanquart S, Necsulea A, Lartillot N, Gouy M (2008) Parallel adaptations to high temperatures in the Archaean eon. Nature 456: 942–945. 19037246 10.1038/nature07393

[67] Chen Y, Cai H, Pan J, Xiang N, Tien P, et al (2009) Functional screen reveals SARS coronavirus nonstructural protein nsp14 as a novel cap N7 methyltransferase. Proc Natl Acad Sci U S A 106: 3484–3489. 19208801 10.1073/pnas.0808790106 PMC2651275

[68] Ivanov KA, Hertzig T, Rozanov M, Bayer S, Thiel V, et al (2004) Major genetic marker of nidoviruses encodes a replicative endoribonuclease. Proc Natl Acad Sci U S A 101: 12694–12699. 15304651 10.1073/pnas.0403127101 PMC514660

[69] Nedialkova DD, Ulferts R, van den Born E, Lauber C, Gorbalenya AE, et al (2009) Biochemical Characterization of Arterivirus Nonstructural Protein 11 Reveals the Nidovirus-Wide Conservation of a Replicative Endoribonuclease. J Virol 83: 5671–5682. 19297500 10.1128/JVI.00261-09 PMC2681944

[70] Decroly E, Imbert I, Coutard B, Bouvet ML, Selisko B, et al (2008) Coronavirus nonstructural protein 16 is a cap-0 binding enzyme possessing (nucleoside-2′O)-methyltransferase activity. J Virol 82: 8071–8084. 18417574 10.1128/JVI.00407-08 PMC2519555

[71] Decroly E, Debarnot C, Ferron F, Bouvet M, Coutard B, et al (2011) Crystal Structure and Functional Analysis of the SARS-Coronavirus RNA Cap 2′-O-Methyltransferase nsp10/nsp16 Complex. PLoS Pathog 7: e1002059. 21637813 10.1371/journal.ppat.1002059 PMC3102710

[72] Putics A, Filipowicz W, Hall J, Gorbalenya AE, Ziebuhr J (2005) ADP-ribose-1″-monophosphatase: a conserved coronavirus enzyme that is dispensable for viral replication in tissue culture. J Virol 79: 12721–12731. 16188975 10.1128/JVI.79.20.12721-12731.2005 PMC1235854

[73] Saikatendu KS, Joseph JS, Subramanian V, Clayton T, Griffith M, et al (2005) Structural basis of severe acute respiratory syndrome coronavirus ADP-ribose-1″-phosphate dephosphorylation by a conserved domain of nsP3. Structure 13: 1665–1675. 16271890 10.1016/j.str.2005.07.022 PMC7126892

[74] Egloff MP, Malet H, Putics A, Heinonen M, Dutartre H, et al (2006) Structural and functional basis for ADP-ribose and poly(ADP-ribose) binding by viral macro domains. J Virol 80: 8493–8502. 16912299 10.1128/JVI.00713-06 PMC1563857

[75] Zanotto PMD, Gibbs MJ, Gould EA, Holmes EC (1996) A reevaluation of the higher taxonomy of viruses based on RNA polymerases. J Virol 70: 6083–6096. 8709232 10.1128/jvi.70.9.6083-6096.1996 PMC190630

[76] Belshaw R, Gardner A, Rambaut A, Pybus OG (2008) Pacing a small cage: mutation and RNA viruses. Trends Ecol Evol 23: 188–193. 18295930 10.1016/j.tree.2007.11.010 PMC7125972

[77] Belshaw R, Pybus OG, Rambaut A (2007) The evolution of genome compression and genomic novelty in RNA viruses. Genome Res 17: 1496–1504. 17785537 10.1101/gr.6305707 PMC1987338

[78] Gorbalenya AE, Koonin EV (1989) Viral-Proteins Containing the Purine NTP-Binding Sequence Pattern. Nucl Acids Res 17: 8413–8440. 2555771 10.1093/nar/17.21.8413 PMC335016

[79] Gorbalenya AE, Koonin EV, Lai MMC (1991) Putative papain-related thiol proteases of positive-strand RNA viruses - Identification of Rubivirus and Aphthovirus Proteases and Delineation of A Novel Conserved Domain Associated with Proteases of Rubivirus, Alpha- and Coronaviruses. FEBS Lett 288: 201–205. 1652473 10.1016/0014-5793(91)81034-6 PMC7130274

[80] Seybert A, Posthuma CC, van Dinten LC, Snijder EJ, Gorbalenya AE, et al (2005) A complex zinc finger controls the enzymatic activities of nidovirus helicases. J Virol 79: 696–704. 15613297 10.1128/JVI.79.2.696-704.2005 PMC538568

[81] Imbert I, Guillemot JC, Bourhis JM, Bussetta C, Coutard B, et al (2006) A second, non-canonical RNA-dependent RNA polymerase in SARS coronavirus. EMBO J 25: 4933–4942. 17024178 10.1038/sj.emboj.7601368 PMC1618104

[82] Zhai YJ, Sun F, Li XM, Pang H, Xu XL, et al (2005) Insights into SARS-CoV transcription and replication from the structure of the nsp7-nsp8 hexadecamer. Nat Struct Mol Biol 12: 980–986. 16228002 10.1038/nsmb999 PMC7096913

[83] Prentice E, McAuliffe J, Lu XT, Subbarao K, Denison MR (2004) Identification and characterization of severe acute respiratory syndrome coronavirus replicase proteins. J Virol 78: 9977–9986. 15331731 10.1128/JVI.78.18.9977-9986.2004 PMC514967

[84] Pan JA, Peng XX, Gao YJ, Li ZL, Lu XL, et al (2008) Genome-Wide Analysis of Protein-Protein Interactions and Involvement of Viral Proteins in SARS-CoV Replication. PLoS One 3: e3299. 18827877 10.1371/journal.pone.0003299 PMC2553179

[85] Imbert I, Snijder EJ, Dimitrova M, Guillemot JC, Lecine P, et al (2008) The SARS-coronavirus PLnc domain of nsp3 as a replication/transcription scaffolding protein. Virus Res 133: 136–148. 18255185 10.1016/j.virusres.2007.11.017 PMC7114086

[86] Nedialkova DD, Gorbalenya AE, Snijder EJ (2010) Arterivirus Nsp1 Modulates the Accumulation of Minus-Strand Templates to Control the Relative Abundance of Viral mRNAs. PLoS Pathog 6: e100772. 10.1371/journal.ppat.1000772 PMC2824749 20174607

[87] Tijms MA, Nedialkova DD, Zevenhoven-Dobbe JC, Gorbalenya AE, Snijder EJ (2007) Arterivirus subgenomic rnRNA synthesis and virion biogenesis depend on the multifunctional nsp1 autoprotease. J Virol 81: 10496–10505. 17626105 10.1128/JVI.00683-07 PMC2045461

[88] Bouvet M, Debarnot C, Imbert I, Selisko B, Snijder EJ, et al (2010) In Vitro Reconstitution of SARS-Coronavirus mRNA Cap Methylation. PLoS Pathog 6: e1000863. 20421945 10.1371/journal.ppat.1000863 PMC2858705

[89] Chen Y, Su CY, Ke M, Jin X, Xu LR, et al (2011) Biochemical and Structural Insights into the Mechanisms of SARS Coronavirus RNA Ribose 2′-O-Methylation by nsp16/nsp10 Protein Complex. PLoS Pathog 7: e1002294. 22022266 10.1371/journal.ppat.1002294 PMC3192843

[90] Belyi VA, Muthukumar M (2006) Electrostatic origin of the genome packing in viruses. Proc Natl Acad Sci U S A 103: 17174–17178. 17090672 10.1073/pnas.0608311103 PMC1859905

[91] Nurmemmedov E, Castelnovo M, Catalano CE, Evilevitch A (2007) Biophysics of viral infectivity: matching genome length with capsid size. Q Rev Biophys 40: 327–356. 18423102 10.1017/S0033583508004666

[92] Krupovic M, Bamford DH (2008) OPINION Virus evolution: how far does the double beta-barrel viral lineage extend? Nat Rev Microbiol 6: 941–948. 19008892 10.1038/nrmicro2033

[93] Zandi R, van der Schoot P (2009) Size Regulation of ss-RNA Viruses. Biophys J 96: 9–20. 18931258 10.1529/biophysj.108.137489 PMC2710049

[94] Chirico N, Vianelli A, Belshaw R (2010) Why genes overlap in viruses. Proc R Soc B 277: 3809–3817. 10.1098/rspb.2010.1052 PMC2992710 20610432

[95] Huang C, Lokugamage KG, Rozovics JM, Narayanan K, Semler BL, et al (2011) Alphacoronavirus Transmissible Gastroenteritis Virus nsp1 Protein Suppresses Protein Translation in Mammalian Cells and in Cell-Free HeLa Cell Extracts but Not in Rabbit Reticulocyte Lysate. J Virol 85: 638–643. 21047955 10.1128/JVI.01806-10 PMC3014157

[96] Kamitani W, Narayanan K, Huang C, Lokugamage K, Ikegami T, et al (2006) Severe acute respiratory syndrome coronavirus nsp1 protein suppresses host gene expression by promoting host mRNA degradation. Proc Natl Acad Sci U S A 103: 12885–12890. 16912115 10.1073/pnas.0603144103 PMC1568942

[97] Neuman BW, Joseph JS, Saikatendu KS, Serrano P, Chatterjee A, et al (2008) Proteornics analysis unravels the functional repertoire of coronavirus nonstructural protein 3. J Virol 82: 5279–5294. 18367524 10.1128/JVI.02631-07 PMC2395186

[98] Tijms MA, van Dinten LC, Gorbalenya AE, Snijder EJ (2001) A zinc finger-containing papain-like protease couples subgenomic mRNA synthesis to genome translation in a positive-stranded RNA virus. Proc Natl Acad Sci U S A 98: 1889–94. 11172046 10.1073/pnas.041390398 PMC29352

[99] Herold J, Siddell SG, Gorbalenya AE (1999) A human RNA viral cysteine proteinase that depends upon a unique Zn2+-binding finger connecting the two domains of a papain-like fold [published erratum appears in J Biol Chem 1999 Jul 23;274(30):21490]. J Biol Chem 274: 14918–14925. 10329692 10.1074/jbc.274.21.14918 PMC8005983

[100] Guan Y, Zheng BJ, He YQ, Liu XL, Zhuang ZX, et al (2003) Isolation and characterization of viruses related to the SARS coronavirus from animals in Southern China. Science 302: 276–278. 12958366 10.1126/science.1087139

[101] Chang HW, de Groot RJ, Egberink HF, Rottier PJM (2010) Feline infectious peritonitis: insights into feline coronavirus pathobiogenesis and epidemiology based on genetic analysis of the viral 3c gene. J Gen Virol 91: 415–420. 19889934 10.1099/vir.0.016485-0

[102] Lorusso A, Decaro N, Schellen P, Rottier PJM, Buonavoglia C, et al (2008) Gain, Preservation, and Loss of a Group 1a Coronavirus Accessory Glycoprotein. J Virol 82: 10312–10317. 18667517 10.1128/JVI.01031-08 PMC2566247

[103] Gorbalenya AE (2008) Genomics and Evolution of the Nidovirales. In: Perlman S, Gallagher T, Snijder EJ, editors. Nidoviruses. Washington: ASM Press. pp. 15–28.

[104] Domingo E (2007) Virus Evolution. In: Knipe DM, Howley PM, Griffin DE, Lamb RA, Martin MA, et al.., editors. Fields Virology. Philadelphia: Wolters Kluwer, Lippincott Williams & Wilkins. pp. 389–421.

[105] Drummond AJ, Rambaut A (2007) BEAST: Bayesian evolutionary analysis by sampling trees. BMC Evol Biol 7: 214. 17996036 10.1186/1471-2148-7-214 PMC2247476

[106] Whelan S, Goldman N (2001) A general empirical model of protein evolution derived from multiple protein families using a maximum-likelihood approach. Mol Biol Evol 18: 691–699. 11319253 10.1093/oxfordjournals.molbev.a003851

[107] Drummond AJ, Ho SYW, Phillips MJ, Rambaut A (2006) Relaxed phylogenetics and dating with confidence. PLoS Biol 4: 699–710. 10.1371/journal.pbio.0040088 PMC1395354 16683862

[108] Yang ZH (2007) PAML 4: Phylogenetic analysis by maximum likelihood. Mol Biol Evol 24: 1586–1591. 17483113 10.1093/molbev/msm088

[109] Felsenstein J (1985) Phylogenies and the Comparative Method. American Naturalist 125: 1–15.

[110] Henikoff S, Henikoff JG (1994) Position-based sequence weights. J Mol Biol 243: 574–578. 7966282 10.1016/0022-2836(94)90032-9

[111] Ramsay JO (1988) Monotone Regression Splines in Action. Statistical Science 3: 425–441.

[112] Lavergne P, Vuong QH (1996) Nonparametric selection of regressors: The nonnested case. Econometrica 64: 207–219.

[113] R Development Core Team (2011) R: A Language and Environment for Statistical Computing. Available: http://www.R-project.org .

[114] van Dinten LC, van Tol H, Gorbalenya AE, Snijder EJ (2000) The predicted metal-binding region of the arterivirus helicase protein is involved in subgenomic mRNA synthesis, genome replication, and virion biogenesis. J Virol 74: 5213–5223. 10799597 10.1128/jvi.74.11.5213-5223.2000 PMC110875

