# Supplementary figures and images for: The Footprint of Genome Architecture in the Largest Genome Expansion in RNA Viruses
Source: PLoS Pathog. 2013 Jul 18;9(7):e1003500. doi: 10.1371/journal.ppat.1003500 (PMC3715407; doi:10.1371/journal.ppat.1003500)

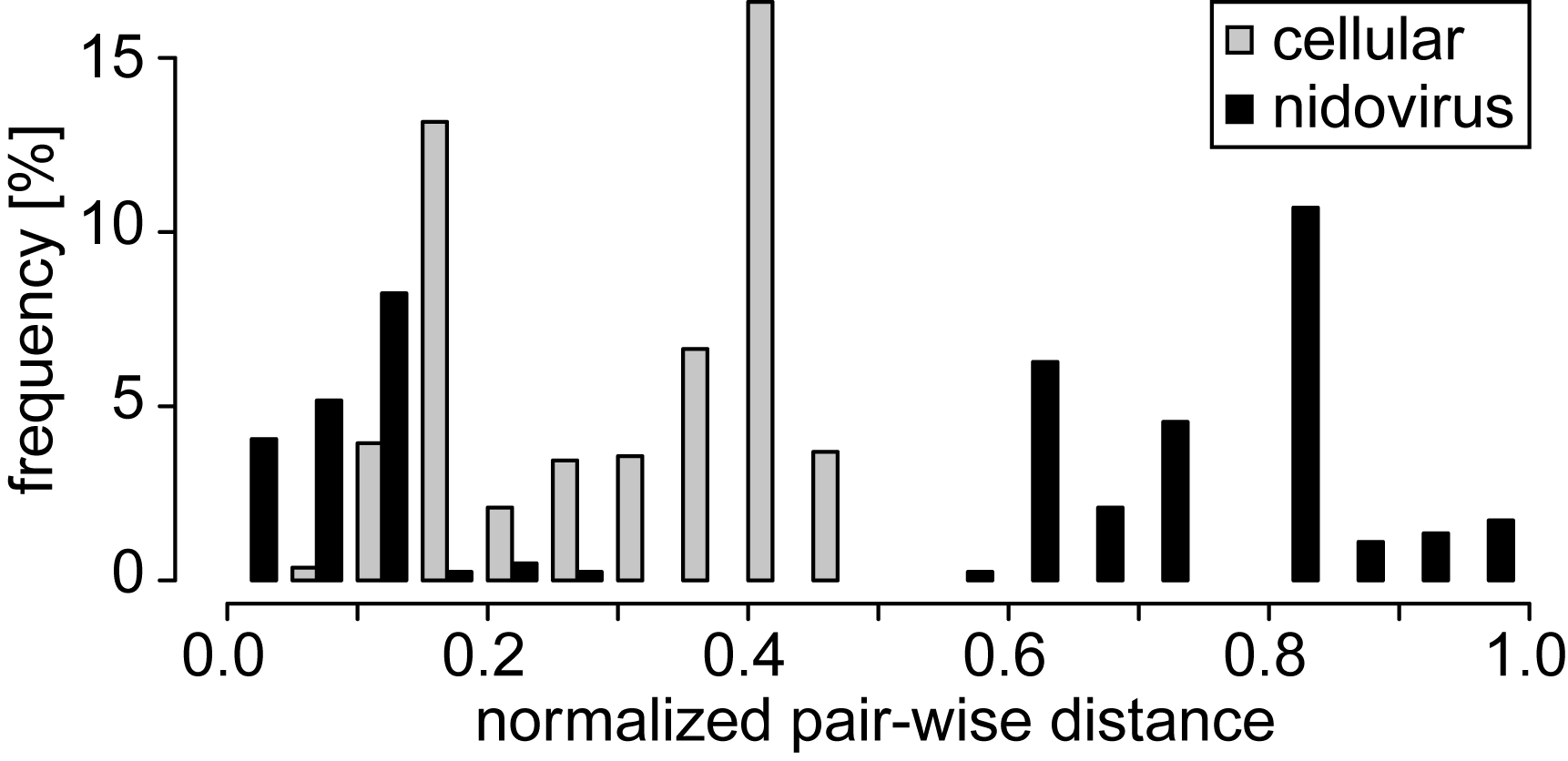

Supplement: Figure S1 — Comparison of genetic distances of nidoviruses and cellular organisms. Shown are the distributions of pair-wise distances for nidovirus and cellular single-copy conserved proteins according to the phylogenies in Fig. 1. The combined set of distances was normalized relative to the largest distance that was set to one. (TIF) [file ppat.1003500.s001.tif]

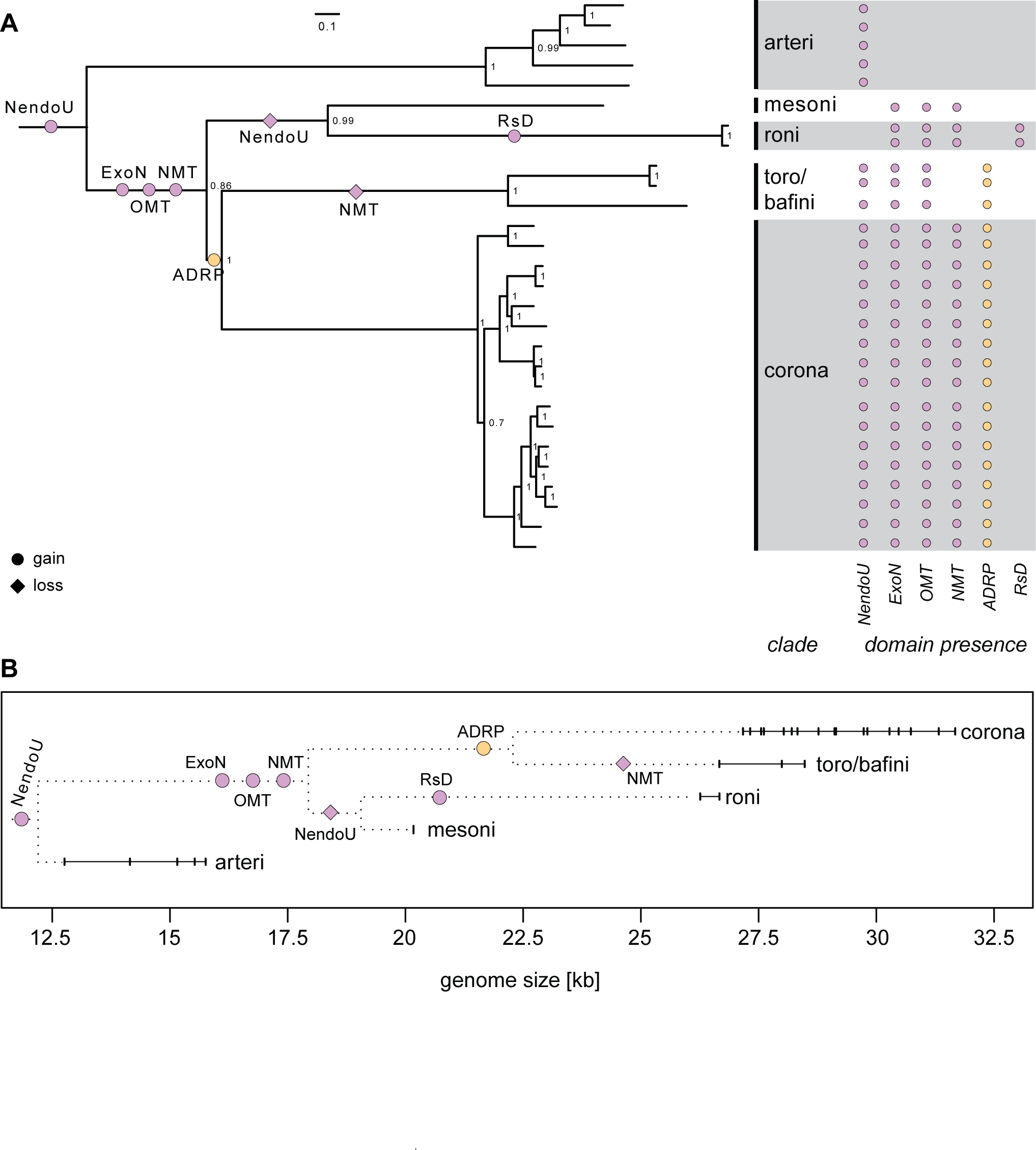

Supplement: Figure S2 — Gain and loss of selected ORF1a/ORF1b domains found in subsets of nidoviruses. (A) Distribution of six selected domains identified in ORF1a (one) and ORF1b (five) conserved in subsets of 28 nidovirus species (right part). One of the ORF1b-encoded domains (RsD) was identified in this study by inspection of the pp1b alignment as a ronivirus-specific insertion (163 aa) that is located between the conserved RdRp and ZmHEL1 domains (see Fig. 3). Colors indicate a domain's ORF location (purple for ORF1b, yellow for ORF1a). The left part shows predicted gain (circles colored according to its ORF location) and loss (colored diamonds) events at internal branches of the nidovirus phylogeny [10]. Nidovirus ancestral domain compositions were reconstructed utilizing a maximum parsimony analysis implemented in PAML4. Support values are shown in Table S2. (B) The nidovirus phylogeny was mapped on the genome size scale (dotted lines). Individual genome sizes of 28 nidovirus species are shown by vertical dashes and the size range within major lineages by horizontal solid lines. Internal nodes in the tree were arbitrarily placed at half the distance of adjacent branching events connecting two lineages while observing the original topology of the phylogeny. Predicted domain gain/loss events are highlighted as in (A). (TIF) [file ppat.1003500.s002.tif]

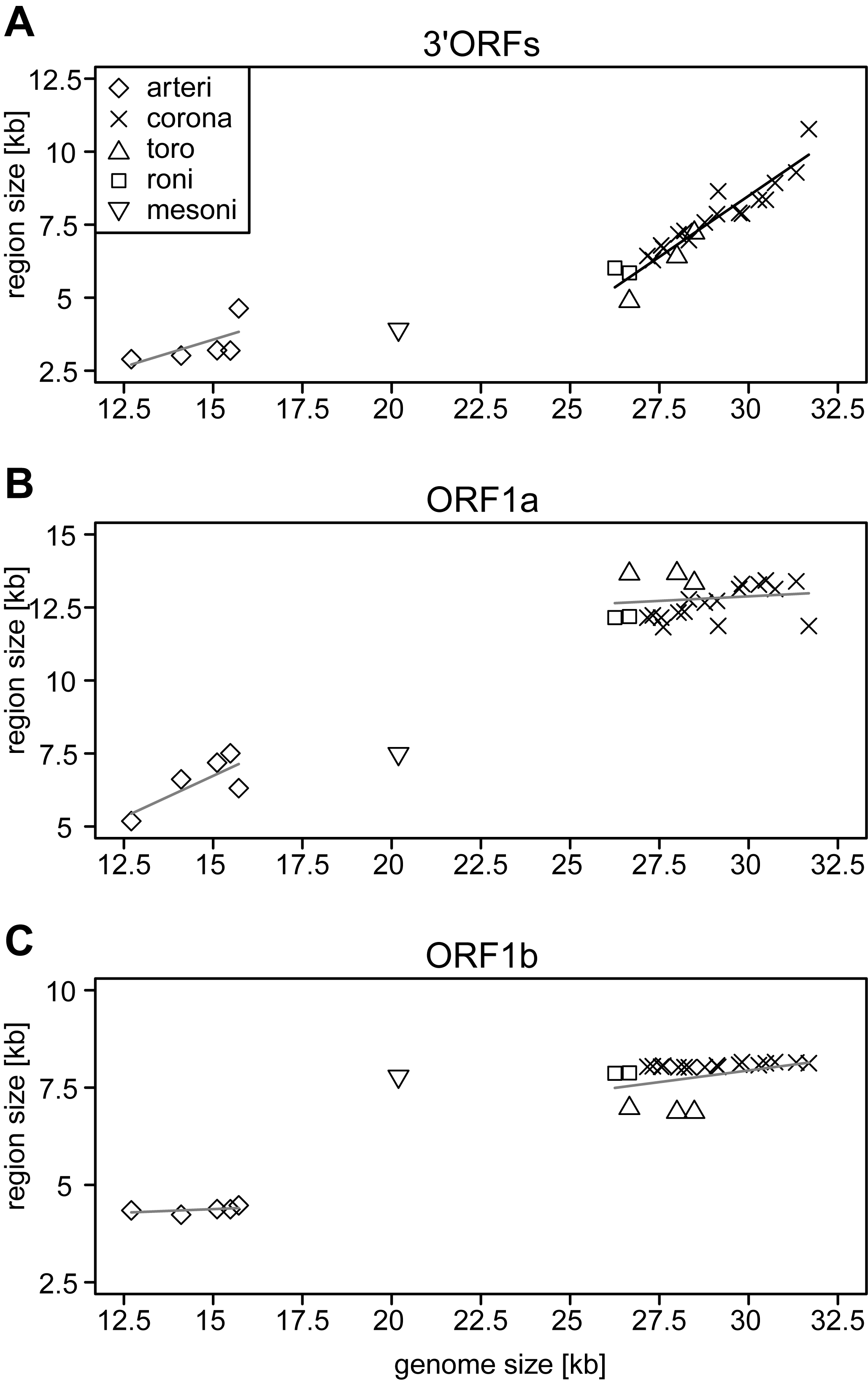

Supplement: Figure S3 — Clade-specific relationship of sizes of three major coding regions and genome size in the nidovirus evolution. For 28 nidoviruses representing species diversity, absolute sizes of 3′ORFs (A), ORF1a (B), and ORF1b (C) are plotted against the size of the genome. Different symbols were used to group the viruses into five major phylogenetic lineages (see inlet in A). Results of weighted linear regression analyses for small-sized (arteri) and large-sized nidoviruses (corona, toro/bafini, roni) are depicted. Regressions with a slope significantly different from zero are shown in black, non-significant ones in grey. The linear regressions fit the data with p = 0.11, r2 = 0.63 (arteri) and p = 0.51, r2 = 0.03 (corona, toro/bafini, roni) for ORF1a, p = 0.34, r2 = 0.30 and p = 0.08, r2 = 0.23 for ORF1b, and p = 0.22, r2 = 0.44 and p = 1e-10, r2 = 0.89 for 3′ORFs. The only significant correlation was observed for 3′ORFs of nidoviruses with large genomes (A) where the regression line showed a slope of 0.84 (±0.07 s.e.). (TIF) [file ppat.1003500.s003.tif]

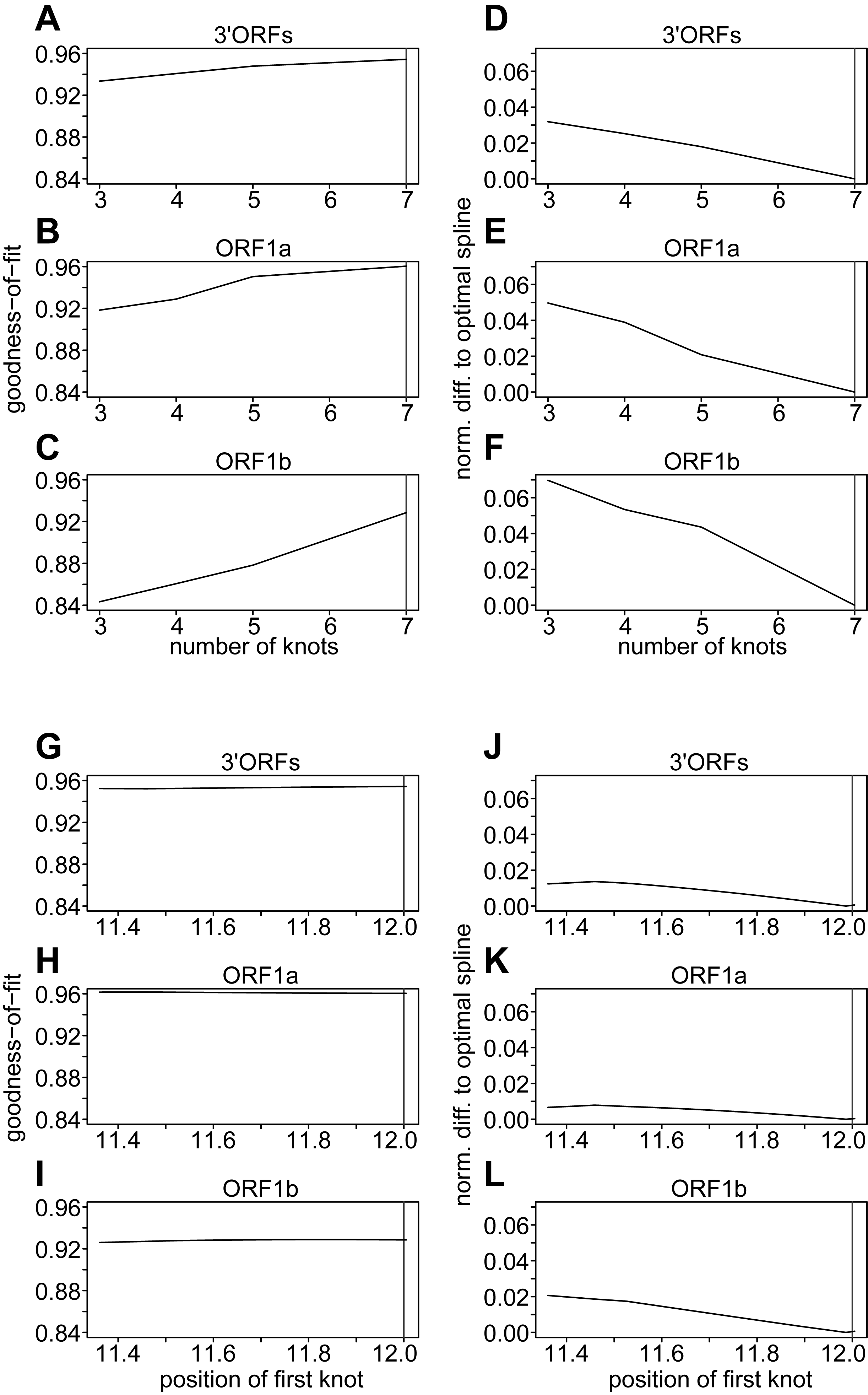

Supplement: Figure S4 — Sensitivity of the splines regression model to the number of knots and the position of the first knot. Shown are goodness-of-fit in form of weighted r2 values (A–C, G–I) and sensitivity on the resulting regression curve (D–F, J–L) for different number of knots in the range of 3 to 7 (A–F) and different positions of the first knot (G–L) for the 3′ORFs, ORF1a and ORF1b genome regions. The best fit was obtained for the 7-knot configuration for all three regions (A–C). Hence, the 7-knot configuration was selected as the optimal one. We have also calculated a difference between other splines models compared to the optimal knot number by calculating the absolute difference of the regression curves of two configurations normalized to the size range of observed values (e.g. size ranges of ORF1a, ORF1b or 3′ORFs). This difference was in the range of 1–7% and increased with decreasing knot number in all three regions (D–F); it could be viewed as the loss of fit relative to the 7-knot configuration. Also, we calculated the model dependence on the position of the first knot by evaluating all positions that do not result in empty bins for the 7-knot configuration, which was found to be in the range from 11.4 to 12.0 kb (G–I). There was virtually no dependence of the position of the first knot and the goodness-of-fit (G–L); we selected the position that is closest to the minimal genome size. The knot number (k = 7) and position of the first knot (at 12 kb resulting in a knot distance of 3.7 kb) used in the main calculation are indicated by green vertical lines. (TIF) [file ppat.1003500.s004.tif]

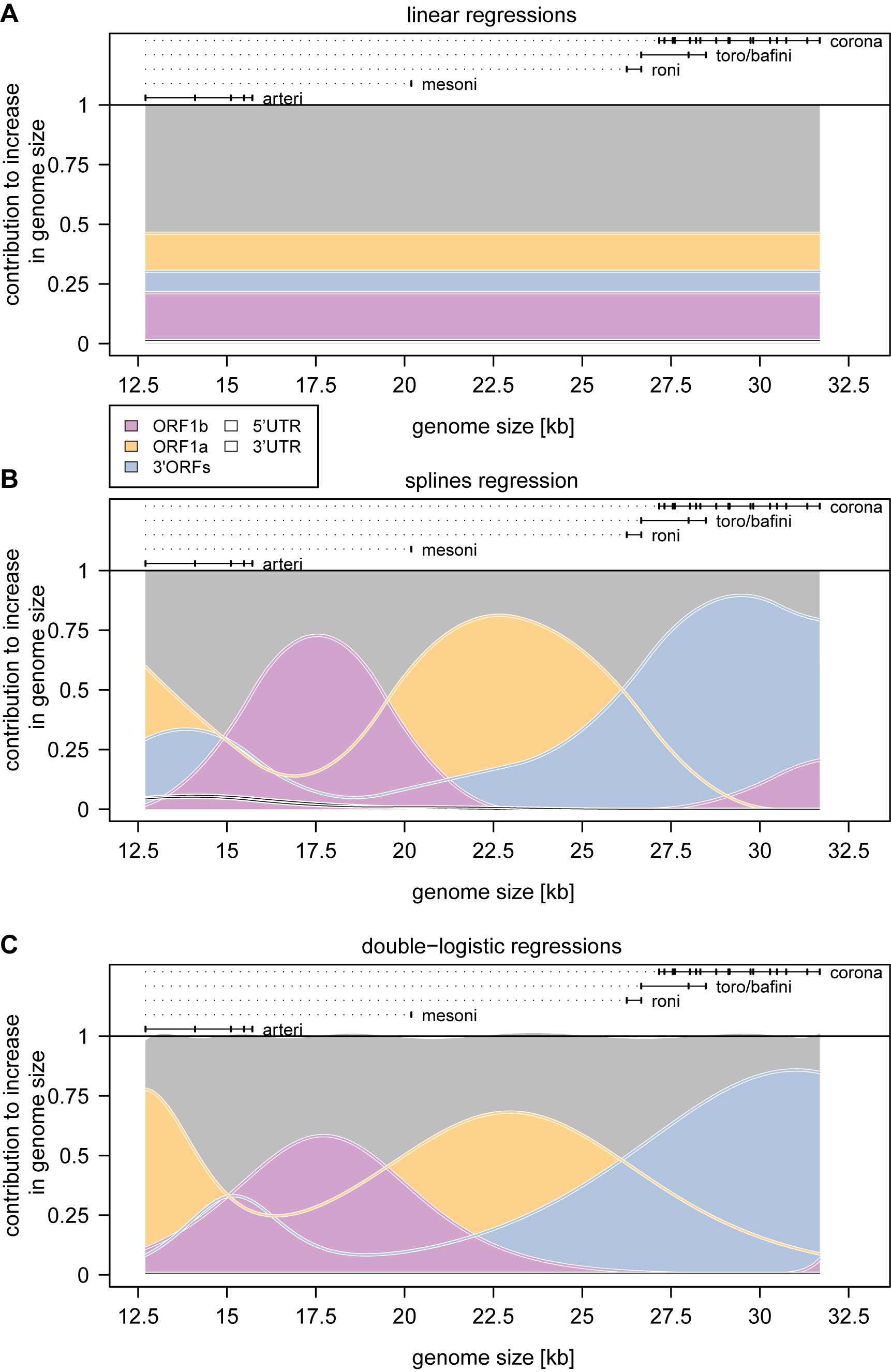

Supplement: Figure S5 — Modeling contribution of ORF1a, ORF1b, 3′ORFs, 5′UTR and 3′UTR to the nidovirus genome expansion. Relative contributions of ORF1a (yellow), ORF1b (purple), 3′ORFs (blue), and 5′ and 3′UTR (black) to the increase in genome size are plotted on top of each other and against their sum = 1 (grey) for the linear (A), the splines (B) and the doublelogistc (C) regression model. Relative size contributions were calculated based on the regression curves fitted to the five genome parts for a dataset of 28 nidoviruses representing species diversity. Solid horizontal lines and vertical bars on top: genome size ranges and virus samplings for arteri-, corona-, toro-/bafini-, roni- and mesoniviruses. Under the linear model (which was statistically rejected in favor of the non-linear models), the contribution of each region to the genome size change is constant by definition. The ORF1a region accounts for most change (46.3%), followed by 3′ORFs (30.2%), ORF1b (21.3%), 5′UTR (1.3%) and 3′UTR (0.9%). In contrast, the splines and double-logistic models predict a cyclic pattern of overlapping wavelike increases of sizes for the three ORFs regions, with maximal contributions of 72.9%, 81.3% and 89.6% for ORF1b, ORF1a and 3′ORFs, respectively (see also main text). Highly similar cyclic and wave-like patterns of region expansions are predicted by the double-logistic model that mostly differs in the amplitude and range of waves compared to those of the splines model. These similarities suggest that the double-logistic model might be an approximation of the monotone splines model facilitating biologically meaningful interpretations. (TIF) [file ppat.1003500.s005.tif]
