# Supplementary material for: The Footprint of Genome Architecture in the Largest Genome Expansion in RNA Viruses
Source: PLoS Pathog. 2013 Jul 18;9(7):e1003500. doi: 10.1371/journal.ppat.1003500 (PMC3715407; doi:10.1371/journal.ppat.1003500)
Supplement: Table S1 — Nidovirus representatives. (DOC) [file ppat.1003500.s006.doc]

**Table S1.** Nidovirus representatives.

| virus |  | virus abbreviation^a^ | (sub)family | accession^b^ |
| --- | --- | --- | --- | --- |
| Nam Dinh virus |  | NDiV_01-03 | Mesoniviridae | DQ458789 |
| Gill-associated virus |  | GAV_96 | *Roniviridae* | AF227196 |
| Yellow head virus |  | YHV_98 | *Roniviridae* | EU487200 |
| White bream virus |  | WBV-DF24_00 | *Torovirinae* | NC_008516 |
| Equine torovirus |  | EToV-Berne_72 | *Torovirinae* | X52374 |
| Bovine torovirus |  | BToV-Breda1_79 | *Torovirinae* | NC_007447 |
| Human coronavirus 229E |  | HCoV-229E_65 | *Coronavirinae* | NC_002645 |
| Human coronavirus NL63 |  | HCoV-NL63_02 | *Coronavirinae* | DQ445911 |
| Miniopterus bat coronavirus 1 |  | Mi-BatCoV-1A_05 | *Coronavirinae* | NC_010437 |
| Rhinolophus bat coronavirus HKU2 |  | Rh-BatCoV-HKU2_06 | *Coronavirinae* | NC_009988 |
| Miniopterus bat coronavirus HKU8 |  | Mi-BatCoV-HKU8_05 | *Coronavirinae* | NC_010438 |
| Scotophilus bat coronavirus 512 |  | Sc-BatCoV-512_05 | *Coronavirinae* | DQ648858 |
| Porcine epidemic diarrhoea virus |  | PEDV-CV777_77 | *Coronavirinae* | NC_003436 |
| Feline coronavirus |  | FCoV_79 | *Coronavirinae* | NC_007025 |
| SARS coronavirus |  | SARS-HCoV_03 | *Coronavirinae* | AY345988 |
| Tylonycteris bat coronavirus HKU4 |  | Ty-BatCoV-HKU4_04 | *Coronavirinae* | EF065505 |
| Pipistrellus bat coronavirus HKU5 |  | Pi-BatCoV-HKU5_04 | *Coronavirinae* | EF065509 |
| Rousettus bat coronavirus HKU9 |  | Ro-BatCoV-HKU9_05 | *Coronavirinae* | EF065513 |
| Human coronavirus HKU1 |  | HCoV-HKU1_04 | *Coronavirinae* | AY884001 |
| Human coronavirus OC43 |  | HCoV-OC43_67 | *Coronavirinae* | AY585228 |
| Mouse hepatitis virus |  | MHV-A59_59 | *Coronavirinae* | AY700211 |
| Infectious bronchitis virus |  | IBV-Beaud_35 | *Coronavirinae* | NC_001451 |
| Beluga whale coronavirus SW1 |  | BWCoV-SW1_06 | *Coronavirinae* | EU111742 |
| Equine arteritis virus |  | EAV-CW_96 | *Arteriviridae* | AY349167 |
| Simian hemorrhagic fever virus |  | SHFV_64 | *Arteriviridae* | NC_003092 |
| Lactate dehydrogenase-elevating virus |  | LDV-P_71 | *Arteriviridae* | U15146 |
| Porcine respiratory and reproductive syndrome virus, North American type |  | PRRSV-NA_95 | *Arteriviridae* | AF176348 |
| Porcine respiratory and reproductive syndrome virus, European type |  | PRRSV-LV_91 | *Arteriviridae* | M96262 |

^a^ acronym of virus name joined (“_”) with sampling year or period for this virus

^b^ Genbank/Refseq accession number
