# Supplementary material for: The Footprint of Genome Architecture in the Largest Genome Expansion in RNA Viruses
Source: PLoS Pathog. 2013 Jul 18;9(7):e1003500. doi: 10.1371/journal.ppat.1003500 (PMC3715407; doi:10.1371/journal.ppat.1003500)
Supplement: Table S2 — Nidovirus ancestral protein domain reconstruction. (DOC) [file ppat.1003500.s007.doc]

**Table S2.** Nidovirus ancestral protein domain reconstruction.

|  | protein domain^b^ | | | | | | | | | | | |
| --- | --- | --- | --- | --- | --- | --- | --- | --- | --- | --- | --- | --- |
| ancestral node^a^ | NendoU | | ExoN | | OMT | | NMT | | ADRP | | RsD | |
| nido (root) | 1 | 1.000 | 0 | 0.576 | 0 | 0.576 | 0 | 0.645 | 0 | 1.000 | 0 | 1.000 |
| arteri | 1 | 1.000 | 0 | 1.000 | 0 | 1.000 | 0 | 1.000 | 0 | 1.000 | 0 | 1.000 |
| large nido+mesoni | 1 | 1.000 | 1 | 1.000 | 1 | 1.000 | 1 | 0.836 | 0 | 1.000 | 0 | 1.000 |
| mesoni+roni | 0 | 1.000 | 1 | 1.000 | 1 | 1.000 | 1 | 1.000 | 0 | 1.000 | 0 | 1.000 |
| roni | 0 | 1.000 | 1 | 1.000 | 1 | 1.000 | 1 | 1.000 | 0 | 1.000 | 1 | 1.000 |
| corona+toro | 1 | 1.000 | 1 | 1.000 | 1 | 1.000 | 1 | 0.836 | 1 | 1.000 | 0 | 1.000 |
| toro | 1 | 1.000 | 1 | 1.000 | 1 | 1.000 | 0 | 1.000 | 1 | 1.000 | 0 | 1.000 |
| corona | 1 | 1.000 | 1 | 1.000 | 1 | 1.000 | 1 | 1.000 | 1 | 1.000 | 0 | 1.000 |

^a^ abbreviations: nidoviruses (nido), large and intermediate size nidoviruses (large nido), roniviruses (roni), mesoniviruses (mesoni), toro-/bafiniviruses (toro), coronaviruses (corona), arteriviruses (arteri).

^b^ shown are the reconstructed state (presence, 1, or absence, 0) and its accuracy by decimal numbers in the range of [0.500-1.000]) at the respective ancestral node for six domains in a maximum parsimony analysis using PAML.
