# Supplementary material for: The Footprint of Genome Architecture in the Largest Genome Expansion in RNA Viruses
Source: PLoS Pathog. 2013 Jul 18;9(7):e1003500. doi: 10.1371/journal.ppat.1003500 (PMC3715407; doi:10.1371/journal.ppat.1003500)
Supplement: Table S3 — Dataset of region and genome sizes used in this study. (RTF) [file ppat.1003500.s008.rtf]

Table S3. Dataset of region and genome sizes used in this study.
		size in nucleotides	
virus	(sub)family	5'UTR	ORF1a	ORF1b	3'ORFs	3'UTR	genome	
NDiV	mesoni	360	7491	7787	3914	640	20192	
GAV	roni	68	12153	7868	6017	147	26253	
YHV	roni	71	12189	7877	5848	677	26662	
WBV	toro	905	13650	6968	4877	260	26660	
EToV	toro	859	13665	6869	6402	197	27992	
BToV	toro	858	13332	6869	7219	201	28479	
HCoV-229E	corona	292	12228	8048	6287	462	27317	
HCoV-NL63	corona	286	12153	8036	6788	287	27550	
BatCoV-1A	corona	271	12777	8021	6970	287	28326	
BatCoV-HKU2	corona	296	12150	8033	6428	258	27165	
BatCoV-HKU8	corona	269	12666	8024	7575	239	28773	
BatCoV-512	corona	293	12357	8024	7286	243	28203	
PEDV	corona	296	12324	8021	7169	223	28033	
FCoV	corona	311	11868	8030	8641	297	29147	
SARS-CoV	corona	249	13134	8087	7903	363	29736	
BatCoV-HKU4	corona	266	13284	8075	8343	318	30286	
BatCoV-HKU5	corona	260	13425	8123	8353	321	30482	
BatCoV-HKU9	corona	228	12723	8069	7862	232	29114	
HCoV-HKU1	corona	199	13305	8153	7877	281	29815	
HCoV-OC43	corona	209	13131	8156	8929	316	30741	
MHV	corona	210	13392	8144	9288	301	31335	
IBV	corona	528	11826	8063	6685	506	27608	
BWCoV	corona	523	11865	8126	10771	401	31686	
EAV	arteri	225	5184	4346	2894	59	12708	
SHFV	arteri	209	6312	4475	4634	87	15717	
LDV	arteri	156	6615	4235	3018	80	14104	
PRRSV-NA	arteri	189	7506	4376	3189	223	15483	
PRRSV-LV	arteri	221	7185	4379	3199	127	15111	
