# Supplementary material for: The Footprint of Genome Architecture in the Largest Genome Expansion in RNA Viruses
Source: PLoS Pathog. 2013 Jul 18;9(7):e1003500. doi: 10.1371/journal.ppat.1003500 (PMC3715407; doi:10.1371/journal.ppat.1003500)
Supplement: Text S1 — Division of labor in the nidovirus genome. (DOCX) [file ppat.1003500.s009.docx]

**Supplementary Text S1**

*Division of labor in the nidovirus genome*

It is noteworthy that the three ORF regions are of similar size but differ in expression mechanism (Fig. 3 top) and principal function. Specifically, ORF1a is the first to be expressed by translation of the incoming virion RNA and, additionally, it encodes the 3CLpro that mediates the release of mature proteins from the polyproteins pp1a and pp1ab. The expression of ORF1b, that follows, depends on the ORF1a region in three different ways: (i) the utilization of ribosomes that have started translation at the ORF1a initiation codon; (ii) the use of the ORF1a/ORF1b RFS shifty codon located upstream of the ORF1a termination codon; and (iii) the proteolytic activity of the ORF1a-encoded 3CLpro. Finally, the expression of the 3’ORFs depends on the products of both ORF1a and ORF1b to form the functional RTC for synthesizing the subgenomic mRNAs that are translated to produce the 3’ORF-encoded proteins [1]. Thus, ORF1a dominates directly and indirectly the expression of the entire genome. On the other hand, ORF1b encodes the principal enzymes for RNA synthesis, e.g. RdRp, and thus dominates the control of replication, while the 3’ORFs encode the components of the virus particles that are the principal vehicles for genome dissemination. This association of the three major processes in the virus life cycle – replication, expression, and dissemination – with three separate regions in the nidovirus genome may be described as a division of labor [2].

**Reference List**

1. Sawicki SG, Sawicki DL, Siddell SG (2007) A contemporary view of coronavirus transcription. J Virol 81: 20-29.

2. Szathmary E, Smith JM (1995) The Major Evolutionary Transitions. Nature 374: 227-232.
