# Supplementary material for: The Footprint of Genome Architecture in the Largest Genome Expansion in RNA Viruses
Source: PLoS Pathog. 2013 Jul 18;9(7):e1003500. doi: 10.1371/journal.ppat.1003500 (PMC3715407; doi:10.1371/journal.ppat.1003500)
Supplement: Text S2 — ADRP acquisition and second wave of genome expansion in coronaviruses. (DOCX) [file ppat.1003500.s010.docx]

**Supplementary Text S2**

*ADRP acquisition may be associated with the second wave of expansion in coronaviruses*

The domain gain/loss description for the second wave is less complete than that for the first wave. Most notable is the acquisition of ADRP (formerly termed “X domain” [1]) which seems to be part of the second wave in large-sized vertebrate nidoviruses represented by viruses of the *Coronaviridae* family (Fig. 6). This domain belongs to the macrodomain protein family with poorly understood function and a broad phyletic distribution in viruses and cellular organisms [2]. The ADRP was shown to have ADP-ribose-1 ''-phosphatase activity [3], to bind poly-ADP-ribose [4], and its inactivation affected cytokine production in coronavirus-infected cells [5]. Although it was proposed to regulate RNA replication [6] and coronavirus pathogenesis [5], its physiological function remains elusive.

**Reference List**

1. Gorbalenya AE, Koonin EV, Lai MMC (1991) Putative papain-related thiol proteases of positive-strand RNA viruses - Identification of Rubivirus and Aphthovirus Proteases and Delineation of A Novel Conserved Domain Associated with Proteases of Rubivirus, Alpha- and Coronaviruses. FEBS Lett 288: 201-205.

2. Pehrson JR, Fuji RN (1998) Evolutionary conservation of histone macroH2A subtypes and domains. Nucl Acids Res 26: 2837-2842.

3. Putics A, Filipowicz W, Hall J, Gorbalenya AE, Ziebuhr J (2005) ADP-ribose-1 ''-monophosphatase: a conserved coronavirus enzyme that is dispensable for viral replication in tissue culture. J Virol 79: 12721-12731.

4. Egloff MP, Malet H, Putics A, Heinonen M, Dutartre H et al. (2006) Structural and functional basis for ADP-ribose and poly(ADP-ribose) binding by viral macro domains. J Virol 80: 8493-8502.

5. Eriksson KK, Cervantes-Barragan L, Ludewig B, Thiel V (2008) Mouse Hepatitis Virus Liver Pathology Is Dependent on ADP-Ribose-1 ''-Phosphatase, a Viral Function Conserved in the Alpha-Like Supergroup. J Virol 82: 12325-12334.

6. Snijder EJ, Bredenbeek PJ, Dobbe JC, Thiel V, Ziebuhr J et al. (2003) Unique and conserved features of genome and proteome of SARS-coronavirus, an early split-off from the coronavirus group 2 lineage. J Mol Biol 331: 991-1004.
